# A standardised method for interpreting the association between mutations and phenotypic drug resistance in *Mycobacterium tuberculosis*

**DOI:** 10.1183/13993003.01354-2017

**Published:** 2017-12-28

**Authors:** Paolo Miotto, Belay Tessema, Elisa Tagliani, Leonid Chindelevitch, Angela M. Starks, Claudia Emerson, Debra Hanna, Peter S. Kim, Richard Liwski, Matteo Zignol, Christopher Gilpin, Stefan Niemann, Claudia M. Denkinger, Joy Fleming, Robin M. Warren, Derrick Crook, James Posey, Sebastien Gagneux, Sven Hoffner, Camilla Rodrigues, Iñaki Comas, David M. Engelthaler, Megan Murray, David Alland, Leen Rigouts, Christoph Lange, Keertan Dheda, Rumina Hasan, Uma Devi K. Ranganathan, Ruth McNerney, Matthew Ezewudo, Daniela M. Cirillo, Marco Schito, Claudio U. Köser, Timothy C. Rodwell

**Affiliations:** 1 Emerging Bacterial Pathogens Unit, IRCCS San Raffaele Scientific Institute, Milan, Italy; 2 Department of Medical Microbiology, University of Gondar, Gondar, Ethiopia; 3 School of Computing Science, Simon Fraser University, Burnaby, BC, Canada; 4 Division of Tuberculosis Elimination, National Center for HIV/AIDS, Viral Hepatitis, STD, and TB Prevention, Centers for Disease Control and Prevention, Atlanta, GA, USA; 5 Institute on Ethics & Policy for Innovation, Department of Philosophy, McMaster University, Hamilton, ON, Canada; 6 Critical Path Institute, Tucson, AZ, USA; 7 Office of AIDS Research, National Institutes of Health, Rockville, MD, USA; 8 Global Tuberculosis Programme, World Health Organization, Geneva, Switzerland; 9 Molecular and Experimental Mycobacteriology, Priority Area Infections, Research Center Borstel, Borstel, Germany; 10 German Center for Infection Research, Borstel, Germany; 11 Foundation for Innovative New Diagnostics, Campus Biotech, Geneva, Switzerland; 12 Key Laboratory of RNA Biology, Institute of Biophysics, Chinese Academy of Sciences, Beijing, China; 13 DST/NRF Centre of Excellence for Biomedical Tuberculosis Research/SAMRC Centre for Tuberculosis Research, Division of Molecular Biology and Human Genetics, Faculty of Medicine and Health Sciences, Stellenbosch University, Stellenbosch, South Africa; 14 Nuffield Department of Medicine, John Radcliffe Hospital, University of Oxford, Oxford, UK; 15 National Infection Service, Public Health England, London, UK; 16 Swiss Tropical and Public Health Institute, Basel, Switzerland; 17 University of Basel, Basel, Switzerland; 18 Microbiology, Tumour and Cell Biology, Karolinska Institute, Stockholm, Sweden; 19 Public Health Agency of Sweden, Solna, Sweden; 20 Hinduja Hospital, Veer Savarkar Marg, Mumbai, India; 21 Tuberculosis Genomics Unit, Biomedicine Institute of Valencia (IBV-CSIC), Valencia, Spain; 22 Foundation for the Promotion of Health and Biomedical Research in the Valencian Community (FISABIO), Valencia, Spain; 23 CIBER (Centros de Investigación Biomédica en Red) in Epidemiology and Public Health, Madrid, Spain; 24 Translational Genomics Research Institute, Flagstaff, AZ, USA; 25 Harvard School of Public Health, Department of Epidemiology, Boston, MA, USA; 26 Center for Emerging Pathogens, Rutgers-New Jersey Medical School, Newark, NJ, USA; 27 Department of Biomedical Sciences, Institute of Tropical Medicine, Antwerp, Belgium; 28 Division of Clinical Infectious Diseases and German Center for Infection Research Tuberculosis Unit, Research Center Borstel, Borstel, Germany; 29 International Health/Infectious Diseases, University of Lübeck, Lübeck, Germany; 30 Department of Medicine, Karolinska Institute, Stockholm, Sweden; 31 Department of Internal Medicine, University of Namibia School of Medicine, Windhoek, Namibia; 32 Lung Infection and Immunity Unit, Department of Medicine, Division of Pulmonology and UCT Lung Institute, University of Cape Town, Groote Schuur Hospital, Cape Town, South Africa; 33 Department of Pathology and Laboratory Medicine, Aga Khan University, Karachi, Pakistan; 34 National Institute for Research in Tuberculosis (ICMR), No 1, Chennai, India; 35 Department of Medicine, Division of Pulmonology, University of Cape Town, Groote Schuur Hospital, Cape Town, South Africa; 36 Department of Genetics, University of Cambridge, Cambridge, UK; 37 Department of Medicine, University of California, San Diego, CA, USA

## Abstract

A clear understanding of the genetic basis of antibiotic resistance in *Mycobacterium tuberculosis* is required to accelerate the development of rapid drug susceptibility testing methods based on genetic sequence.

Raw genotype–phenotype correlation data were extracted as part of a comprehensive systematic review to develop a standardised analytical approach for interpreting resistance associated mutations for rifampicin, isoniazid, ofloxacin/levofloxacin, moxifloxacin, amikacin, kanamycin, capreomycin, streptomycin, ethionamide/prothionamide and pyrazinamide. Mutation frequencies in resistant and susceptible isolates were calculated, together with novel statistical measures to classify mutations as high, moderate, minimal or indeterminate confidence for predicting resistance.

We identified 286 confidence-graded mutations associated with resistance. Compared to phenotypic methods, sensitivity (95% CI) for rifampicin was 90.3% (89.6–90.9%), while for isoniazid it was 78.2% (77.4–79.0%) and their specificities were 96.3% (95.7–96.8%) and 94.4% (93.1–95.5%), respectively. For second-line drugs, sensitivity varied from 67.4% (64.1–70.6%) for capreomycin to 88.2% (85.1–90.9%) for moxifloxacin, with specificity ranging from 90.0% (87.1–92.5%) for moxifloxacin to 99.5% (99.0–99.8%) for amikacin.

This study provides a standardised and comprehensive approach for the interpretation of mutations as predictors of *M. tuberculosis* drug-resistant phenotypes. These data have implications for the clinical interpretation of molecular diagnostics and next-generation sequencing as well as efficient individualised therapy for patients with drug-resistant tuberculosis.

## Abstract 2

**A comprehensive basis for interpreting mutations to predict antibiotic resistance in tuberculosis** http://ow.ly/hhwJ30g9jCY

## Introduction

In 2015, only 20% of the 580 000 people eligible for multidrug-resistant tuberculosis (MDR-TB) treatment received an appropriate drug regimen [1]. Treatment of MDR-TB is long, expensive and toxic; errors in the design of the regimen are associated with increased rates of failure and death [2, 3]. Drug-resistant tuberculosis regimens need to include a sufficient number of effective drugs, a significant challenge for clinicians worldwide, as most are forced to make therapy decisions without any drug susceptibility testing (DST) information. Additionally, the World Health Organization (WHO) policy guidance for the use of novel antituberculosis drugs (bedaquiline and delamanid) and newly developed shorter regimens for the treatment of drug-resistant TB requires rapid diagnosis and triaging of patients to identify those who are most likely to benefit from the new treatment options [4–8]. Phenotypic DST is not suitable for this purpose, as it takes weeks to complete due to the slow growth rate of *Mycobacterium tuberculosis* complex (MTBC) strains, and requires both expensive infrastructure and considerable technical expertise [9]. As targeted genotypic DST assays (that provide results within hours to days) have been shown to be accurate, increasingly automated and cost-effective, they are proving to be a viable alternative or effective complement to phenotypic DST [9]. However, continuing technical constraints of the current molecular assays restrict the number of resistance determinants and genomic regions that can be evaluated, which limit the clinical value of the assays. By contrast, whole-genome sequencing (WGS) has the potential to provide near-complete information as it includes almost the entire genetic repertoire of a given clinical MTBC strain. However, the data analysis is more complex, and in order to maximise clinical utility of WGS, healthcare workers need clear rules to interpret the clinical relevance of genetic changes that are detected [10]. A high-quality and comprehensive catalogue of genetic markers of resistance (*i.e.* mutations that either cause resistance or compensate for resistance) is needed to distinguish significant resistant variants from those that are not. For some drugs this requires a precise understanding of the level of resistance conferred by the mutation in question, which is expressed as a range of minimum inhibitory concentrations (MICs) found in strains harbouring a specific mutation, as well as an understanding of the degree of cross-resistance conferred for antibiotics with shared modes of action [11, 12]. Although *in vitro* allelic exchange experiments prove conclusively that a particular mutation is both necessary and sufficient to confer phenotypic resistance, these approaches are expensive, slow and technically demanding, and they are only suitable to investigate the function of novel resistance genes or, at best, a limited number of resistance mutations per gene [13]. Consequently, *in silico* association studies are indispensable to investigate the vast majority of suspected resistance mutations, particularly in nonessential genes, where hundreds of loss-of-function mutations can result in resistance [14].

Attempts have been made to combine disparate datasets to collect the necessary evidence for these associations. Unfortunately, most of these databases are not actively curated or lack significant clinical metadata [15]. Moreover, these databases focus mainly on collecting and presenting published data, leaving the final interpretation of the genotype–phenotype correlation to the user [16, 17]. More fundamentally, there is no consensus regarding the threshold of evidence required to classify a mutation as a valid marker for phenotypic resistance.

In this study we 1) describe a standardised analytical approach for assessing and quantifying the strength of the association of a particular mutation or group of mutations with phenotypic antibiotic resistance; 2) demonstrate how this approach can be used by applying it to data from the most comprehensive systematic review of MTBC drug resistance mutations conducted to date; and 3) apply the resulting graded mutation list to the interpretation guidelines for the WHO-endorsed, targeted genotypic DST assays Hain GenoType MTBDR*plus* v2.0 and MTBDR*sl* v2.0 [9]. This study has implications for the clinical interpretation of both targeted molecular and WGS-based diagnostics for drug-resistant TB, and is intended to provide clarity and build confidence regarding the genetic basis of resistance in MTBC for both molecular assay developers and the clinicians interpreting those assays.

## Materials and methods

### Data collection

A systematic literature review on the association of sequencing and phenotypic DST data for MTBC was undertaken for selected anti-TB drugs and resistance genes (table 1). Expert consensus from the global ReSeqTB Data Sharing Platform was utilised to define the loci with the highest likelihood of association with resistance, and the review was limited to those loci (online supplementary material 1) [18]. A comprehensive search of the National Center for Biotechnology Information (NCBI) PubMed database for relevant citations was performed; the list of search terms and the data collection form are available as online supplementary material 2 and 3. The quality of the studies included was appraised using a modified Quality Assessment of Diagnostic Accuracy Studies (QUADAS)-2 tool (online supplementary material 4) [19]. The sensitivity and specificity of predicting phenotypic ofloxacin (OFX) and levofloxacin (LFX) resistance by sequencing were found to be independent of the phenotypic method used, whereas there were substantial differences in specificity for moxifloxacin (MFX) resistance prediction, depending on whether liquid or solid DST was used as the reference method (data not shown). Results for OFX and LFX from both testing methods were therefore pooled, whereas MFX results were analysed separately for each DST method. To maximise the number of isolates studied and thus increase statistical power, results for ethionamide (ETO) and prothionamide (PTO) were also pooled.

**TABLE 1:** Overview of the data included in the study

	**Collected data**				**Studies**	
	**Loci of interest**	**Total isolates**	**Isolation time frame years**	**Countries represented**	**Screened**	**Included**
**Rifampicin (R)**	*rpoB*	13 424	1999–2014	37	459	95
**Isoniazid (H)**	*katG*	11 847	1992–2014	42	650	127
	*inhA-mabA*	9407				
	*furA*	361				
	*mshA*	288				
**Ethionamide and prothionamide (ETO/PTO)**	*inhA-mabA*	346				
	*ethA*	181				
	*mshA*	117				
**Ofloxacin (OFX)**	*gyrA*	5911	1991–2013	36	243	75
	*gyrB*	3078				
**Moxifloxacin (MFX)**	*gyrA*	1019				
	*gyrB*	735				
**Levofloxacin (LFX)**	*gyrA*	449				
	*gyrB*	218				
**Pyrazinamide (Z)**	*pncA*	4949	1990–2014	36	378	81
**Streptomycin (S)**	*rpsL*	3263	1985–2013	43	423	104
	*tap*	0				
	*rrs*	2598				
	*whiB7*	0				
	*gidB*	812				
**Amikacin (AM)**	*rrs*	2105				
**Capreomycin (CM)**	*rrs*	2533				
	*tlyA*	1854				
**Kanamycin (KM)**	*rrs*	1727				
	*eis*	2029				
	*whiB7*	56				

Data are presented as n. Inclusion and exclusion criteria for individual studies are reported in online supplementary material 2.

### Development of a standardised methodology for the statistical validation of the association of a mutation with resistance

An expert, consensus-driven approach was used to develop a standardised procedure for grading drug resistance-associated mutations. The collated data were used to calculate the frequency of each mutation in resistant and susceptible MTBC isolates and to derive a likelihood ratio. In this approach, likelihood ratios were used for objectively evaluating whether mutations were positively or negatively associated with phenotypic resistance. Moreover, odds ratios were considered when evaluating the association of the genotypic and phenotypic data. Using this rationale, the thresholds commonly adopted in evidence-based medicine were adapted to grade the MTBC mutations [20–22]. Details of the statistical analysis are provided in online supplementary material 5. Mutations were classified as having either high, moderate or minimal confidence for being associated with resistance, or as indeterminate or “not associated with resistance” (see table 2 for the definitions of each category). The procedure used for nonsense mutations, insertions/deletions and silent mutations is described in detail in online supplementary material 5. Results from the three types of phenotypic reference standards used (liquid, solid and combined-media DST) were compared using a series of rules that are outlined in online supplementary material 5 to yield confidence values for individual mutations (“individual confidence values” (ICVs)), associations with a specific medium (“medium confidence values” (MCVs)) and an overall, best confidence value (BCV). Moreover, interpretive confidence values for each of the aforementioned categories (*i.e.* iICVs, iMCVs and iBCV, respectively) were calculated to extrapolate the confidence values of individual mutations and the pooled results for insertions/deletions and nonsense mutations.

**TABLE 2:** Overview of proposed confidence levels for grading mutations associated with phenotypic resistance

	**Symbol**	**LR^+^ and OR**	
		**p*-*value**	**value**
**High (Hi) confidence for association with resistance** Strong association of the mutation with phenotypic drug resistance; sufficient evidence that the mutation confers or is strongly associated with drug resistance	#•	<0.05	>10
**Moderate (Mo) confidence for association with resistance** Moderate association of the mutation with phenotypic drug resistance; additional data desirable for improved evidence that the mutation confers or is strongly associated with drug resistance	#•	<0.05	5< … ≤10
**Minimal (Mi) confidence for association with resistance** Weak association of the mutation with phenotypic drug resistance; inconclusive evidence that the mutation confers or is strongly associated with drug resistance. Substantial additional data required	#•	<0.05	1< … ≤5
**No association with resistance** No evidence of association between the mutation and drug resistance	#•	<0.05	<1
**Indeterminate** No statistically significant threshold reached; additional data required	Indeter	≥0.05	

The table shows the thresholds applied to likelihood ratios (LR) and odds ratios (OR) to grade the association of mutations with phenotypic drug resistance. LR^+^: positive likelihood ratio. “Additional data” is defined as a requirement for 1) more phenotypically drug resistant and susceptible isolates tested with the mutation in question; and/or 2) better understanding of the mechanism of drug resistance (*e.g.* to investigate epistasis, or the interactions between drug-resistance conferring mutations, lineage-specific genetic factors and compensatory mutations [23, 24] or synergistic factors when more than one mutation is required to confer resistance [25]).

Results were stratified according to the following reference standards for each target-drug combination, given that systematic differences between media have been observed previously. 1) Liquid-media phenotypic DST performed according to WHO guidelines; 2) solid-media phenotypic DST performed according to WHO guidelines (for pyrazinamide (Z), the Wayne enzymatic assay, which is not WHO-endorsed, was also considered in this category); and 3) liquid- and solid-media DST combined (this category included DST results for which the medium used was unclear, which meant that the number in this category was sometimes larger than the sum of categories 1 and 2).

## Results

### Overview of the datasets included in the systematic review

Table 1 summarises the main features of the datasets considered. Data from up to 43 countries and up to 13 424 isolates per gene locus or antimycobacterial drug were included. PRISMA (Preferred Reporting Items for Systematic Reviews and Meta-Analyses) diagrams for each drug and a detailed breakdown of exclusion criteria, types of studies included, global representativeness of datasets and phenotypic DST methods can be found in online supplementary material 6.

The majority of the phenotypically resistant isolates harboured either single point mutations or, more rarely, insertions/deletions in the resistance genes/loci that were studied (mean 82.6%, range 69.8–95.6%; online supplementary material 6, figure S6.20A). Conversely, phenotypically susceptible isolates had mostly wild-type results (mean 85.6%, range 70.9–97.3%; online supplementary material 6, figure S6.20B). However, both frequencies probably represent an underestimate, given that some studies only reported the variants that were thought to be responsible for the resistant phenotype. Well-known nonsynonymous polymorphisms that do not confer resistance, such as *katG* R463L, or synonymous mutations were not always reported [26] (online supplementary material 4).

### Overview of confidence graded mutations

**FIGURE 1:**
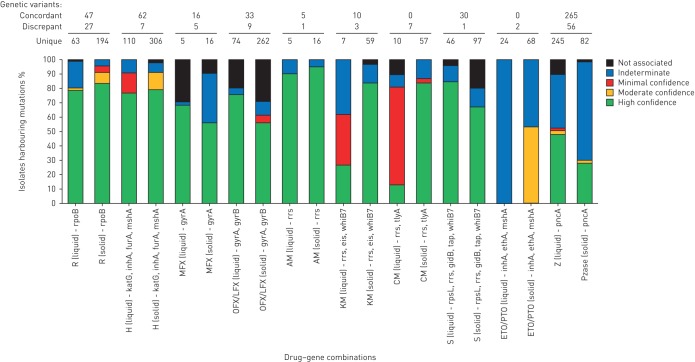
Medium confidence values (MCVs) stratified by confidence value, drug susceptibility testing medium and antibiotic-resistance gene combination. In the three rows above the graph we show variants that were concordant on both media, the number of variants that had different confidence levels on liquid and solid (these are marked as “discrepant variants” and are listed in full in online supplementary material 9) and unique variants for which confidence levels were available on only one of the two media.

A full list of confidence values for associations of ICVs, MCVs or the overall BCVs and their corresponding interpretative confidence values can be found in online supplementary material 7 and 9. Figure 1 provides an overview of the proportion of isolates with different confidence levels for MCVs. For all drugs, data for the majority of variants were only available from one medium (solid or liquid). Where mutations were tested on both media, the MCVs were usually identical. However, there were also variants, for which the confidence levels differed (online supplementary material 9). The number of these discrepancies varied from just one variant for drugs such as amikacin (AM), to 56 for pyrazinamide (Z). Some of these discrepancies were minor (*e.g.* the *gyrA* D94N variant had minimal confidence on solid medium for OFX/LFX, but high confidence for liquid medium), whereas others were major. Important differences included 1) *gyrA* A90V, which was not associated with resistance when the phenotypic reference standard was liquid medium but had a high-confidence MCV for MFX resistance when associated with phenotypic resistance determined in solid media; and 2) *rpoB* L511P, which was not associated with resistance in liquid medium, but had minimal confidence on solid medium. In order to be conservative in the interpretation of these cases, the lower statistical confidence was overruled by the higher MCV to yield a BCV (see online supplementary material 5 for more details). Similarly, MCVs for which evidence was available on one medium only were used as the BCVs.

### Overview of BCVs

Table 3 lists all of the 394 MTBC genetic variants with high, moderate (192 mutations plus 202 frameshifts and premature stop codons) or minimal BCVs, as well as 40 changes that were found not to be associated with resistance according to nominal p*-*values (online supplementary material 5). Six of these variants identified with our association method had to be graded as not associated with phenotypic resistance manually based on expert knowledge. For example, the a514c and c517t mutations in *rrs* had high-confidence BCVs for predicting kanamycin (KM) resistance, but were excluded from further analysis because there was no known causative link between these mutations and KM resistance [27, 28]. Other mutations (*e.g. inhA* g-102a) were excluded as they are known markers for particular MTBC genotypes (lineage or sublineages) and do not confer resistance [26, 29]. We highlighted the 286 variants with high, moderate or minimal BCVs (111 mutations plus 150 frameshifts and 25 premature stop codons), as well as 18 changes that were found to be “not associated” with resistance (likelihood ratio <1) that remained statistically significant after correcting the p-value for the false discovery rate (see online supplementary material 5 for details). The resulting subset of 304 BCVs is referred to as the corrected BCVs for the remainder of this article. Overall, we identified 286 confidence-graded (high + moderate + minimal (Hi+Mo+Mi)) mutations associated with phenotypic resistance.

**TABLE 3:** List of confidence-graded mutations associated with phenotypic drug resistance as determined by best confidence values

**Drug (phenotypic testing)**	**Gene**	**High-confidence mutations**	**Moderate-confidence mutations**	**Minimal-confidence mutations**	**No association with resistance**
**First-line**	* *				
Rifampicin (R)	*rpoB*	F505V+D516Y, S512T, Q513H+L533P, Q513-F514ins, **Q513K**, **Q513L**, **Q513P**, **F514dupl,** M515I+D516Y, **D516A**, **D516F**, **D516G**, D516G+L533P, D516ins, D516N, **D516V,** Del N518, **S522Q**, **H526C**, **H526D**, H526F, **H526G**, **H526L**, **H526R**, **H526Y S531F**, **S531L**, S531Q, **S531W**, S531Y, D626E	**D516Y**, **S522L**, H526P, **L533P**	**L511P**, **H526N**, **I572F**	
Isoniazid (H)	*inhA-mabA*	g-102a^#,¶^	**c-15t**		**g-102a**^#,¶^, t-80g, *g-47c*, **T4I**
	*katG*	S315I, **S315N**, **S315T**, pooled frameshifts and premature stop codons			A110V, **R463L**, L499M
	*furA*				**L68F**
	*mshA*		A187V^#,¶^		**N111S**
**Second-line (group A)**	* *				
Moxifloxacin (MFX)	*gyrA*	G88C, **A90V**, **S91P**, **D94A**, **D94G**, **D94N**, **D94Y**			E21Q, **S95T**, G247S, G668D, V712L
Ofloxacin (OFX)/levofloxacin (LFX)	*gyrA*	**G88A**, **G88C**, **S91P**, **A90V**, **D94A**, **D94G**, **D94H**, **D94N**, **D94Y**	D89N		E21Q, **T80A**, **S95T**, G247S, G668D, V712L
	*gyrB*	E459K, **A504V**			
**Second-line (group B)**	* *				
Amikacin (AM)	*rrs*	**a1401g**, **g1484t**			
Kanamycin (KM)	*eis*	**c-14t**, **g-10a**		g-37t, c-12t	**a1338c**
	*rrs*	a514c^#^, **a1401g**, c1402t, g1484t			
	*rrs+eis*	*rrs* c517t^#^ + *eis* g-37t			
Capreomycin (CM)	*rrs*	**a1401g**, **c1402t**, **g1484t**			**c517t**
	*tlyA*	**N236K**, p**ooled frameshifts and premature stop codons**			D149H
Streptomycin (S)	*rpsL*	**K43R**, K43T, **K88Q**, **K88R**, T40I			
	*rrs*	a1401g^#^, **a514c**, a514t, c462t, c513t, **c517t**			
	*gidB*		**E92D**^#,¶^		**L16R**, **V110G**, pooled frameshifts and premature stop codons
**Second-line (group C)**	* *				
Ethionamide and prothionamide (ETO/PTO)	*inhA*	c-15t+I194T, c-15t+S49A	**c-15t**		
	*ethA*				Q347Stop
**Second-line (group D)**	* *				
Pyrazinamide (Z)	*pncA*	t-12c, **a-11g**, t-7c, **A3E**, L4S, **I6T**, V7G, D8E, **D8G**, D8N, **Q10P**, **D12A**, **D12N**, **C14R**, G17D, L19P, G24D, **Y34D**, **A46V**, **K48T**, **D49G**, D49N, **H51Q**, H51R, P54S, **H57D**^¶^, H57P, **H57R**, **H57Y**, S59P, P62L, P62Q, **D63G**, S66P, **S67P**, W68C, **W68R**, **H71D**, H71Q, **H71Y**, **C72R**, **T76P**, **H82R**, **L85P**, **L85R**, **F94L**, **F94S**, K96N, K96R, G97C, **G97D**, **G97S**, **Y103H**, **S104R**, **G108R**, L116P, **L116R**, **L120P**, R123P, V125F, **V125G**, **V128G**, G132A, G132D, G132S, A134V, **T135N**, **T135P**, H137P, C138Y, **V139G**, **V139L**, **Q141P**, **T142A**, **T142K**, **T142M**, **indel - R148ins (inframe)**, L151S, **V155G**, L159P, T160P, G162D, T168P, **L172P**, M175T, **M175V**, V180F, V180G, **Pooled frameshifts and premature stop codons**	**V7G**, Q10R, **P54L**, **W68G**, K96E, K96T, A171E, M175I	**D12G**, F58L, H71R, I133T, **V139A**	**indel - c-125del**, **I31T**, **L35R**, **T47A**, **I6L**, K48T, T114M

The table includes all the mutations graded according to the proposed standardised approach for providing confidence levels to their association with phenotypic drug resistance. Standard type represents associations based on nominal p-values (putative); bold type represents associations based on corrected p*-*values. The rationale for pooling insertions/deletions and nonsense mutations can be found in online supplementary material 5. Tables 1 and 2 provide the details of the data included in the grading system and the definitions for the confidence categories. Indeterminate mutations were not included in the table and can be found in online supplementary material 8. Drugs were classified based on the updated guidelines for short and individualised regimens [4]. ^#^: six associations were not considered for further analysis as there was probably no causative relationship between these genetic changes and the resistance to the antibiotic in question; ^¶^: genotype-specific mutation.

### Diagnostic performance of corrected iBCVs

Online supplementary material 10 provides a comprehensive overview of the performance characteristics (sensitivity, specificity and diagnostic accuracy) for different categories of corrected iBCVs (see online supplementary material 5 for a detailed explanation of the differences between BCV and iBCV): 1) high, moderate and minimal confidence mutations individually; 2) Hi+Mo+Mi confidence mutations combined; 3) indeterminate (I) mutations; and 4) a combination of Hi+Mo+Mi+I confidence mutations as well as mutations that are “not associated with phenotypic resistance” (referred herewith as “all mutations”).

**FIGURE 2:**
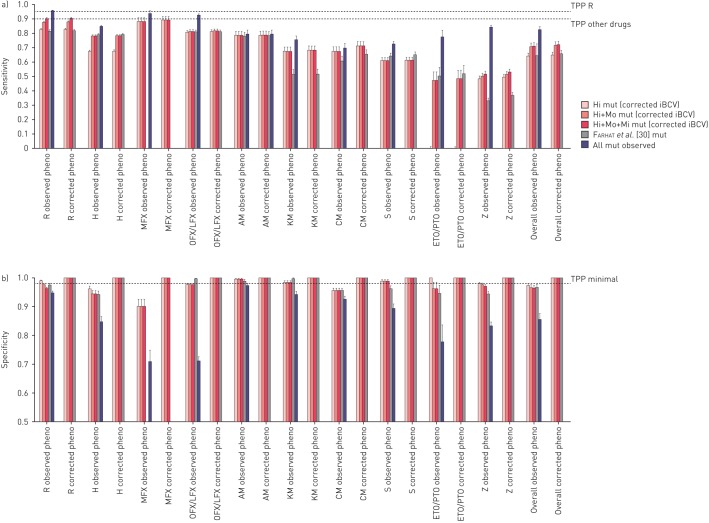
Comparison of the sensitivity and specificity of different groups of mutations. For each drug, two types of comparison were performed. First, the a) sensitivities and b) specificities were calculated with the associated 95% confidence levels for the “observed” phenotypic result. Specifically, the figures for high (Hi), high and moderate (Hi+Mo) and high and moderate and minimal (Hi+Mo+Mi) confidence interpretative best confidence values (iBCVs) were compared with using all mutations observed in the study. Moreover, genetic variants were included from a recent study by Farhat *et al.* [30]. Second, we conducted the same comparison using a “corrected” phenotype as reference (*i.e.* where we assumed that strains that were phenotypically susceptible but harboured either a Hi, Hi+Mo or H+Mo+Mi confidence iBCV mutation or mutations by Farhat *et al*. were false-susceptible results). For some drugs, such as capreomycin (CM), both percentages remained unchanged as all mutations were high confidence. Minimal target product profile (TPP) thresholds set by the World Health Organization for new molecular-based diagnostic tools compared to phenotypic drug susceptibility testing [31] are shown. These were intended for rifampicin (R), isoniazid (H), fluoroquinolones, kanamycin (KM), amikacin (AM) and CM only. However, in addition we included the threshold for the remaining drugs and overall results for comparison (for additional details see online supplementary material 11). MFX: moxifloxacin; OFX: ofloxacin; LFX: levofloxacin; S: streptomycin; ETO/PTO: ethionamide and prothionamide; Z: pyrazinamide.

The sensitivities (95% CI) of mutations with high-confidence corrected iBCVs compared with phenotypic DST (*i.e.* the observed resistant phenotype) ranged from 0.0% (0.0–0.01%) for ETO/PTO to 88.2% (85.1–90.9%) for MFX (figure 2a). Specificities (95% CI) varied from 95.6% (94.7–96.4%) for capreomycin (CM) to 99.5% (99.0–99.8%) for AM. The inclusion of Mo and Mo+Mi confidence mutations resulted in a gain in sensitivity of 0–47.3% with only marginal decreases in specificity (*i.e.* 0–3.8%). The performance of the Hi+Mo+Mi confidence mutations identified in this study performed as well or better than a set of diagnostic mutations recently proposed by Farhat *et al.* [30] that were based on detecting resistance-associated mutations using random forest modelling on a set of 1400 MTBC isolates (online supplementary material 11, table S11.1).

Assuming that mutations with high, moderate and minimal confidence corrected iBCVs are true markers or resistance, a “corrected phenotype” was calculated for each drug (*i.e.* the sum of the phenotypically resistant isolates and phenotypically susceptible isolates with one of the aforementioned mutations). Accordingly, the proportion of resistance missed by phenotypic DST ranged from 0.7% (95% CI 0.3–1.6%) for AM to 11.9% (95% CI 9.8–14.1%) for CM, which resulted in a difference of 0.2% and 3.9%, respectively, between the sensitivities using the “corrected phenotype” as the reference standard *versus* phenotypic DST (the specificities became, by definition, 100%) (table 4 and figure 2b).

**TABLE 4:** Overview of phenotypically susceptible isolates with high (Hi), moderate (Mo) or minimal (Mi) confidence corrected interpretative best confidence values (iBCVs)

**Drug**	**Drug-resistant phenotype**	**Hi confidence iBCVs**			**Hi+Mo confidence iBCVs**			**Hi+Mo+Mi confidence iBCVs**		
		**False-susceptible**	**Resistance missed % (95% CI)**	**Difference in sensitivity %**	**False-susceptible**	**Resistance missed % (95% CI)**	**Difference in sensitivity %**	**False-susceptible**	**Resistance missed % (95% CI)**	**Difference in sensitivity %**
**Rifampicin (R)**	8294	55	0.7 (0.5–0.9)	0.1	124	1.5 (1.2–1.8)	0.2	192	2.3 (2.0–2.6)	0.2
**Isoniazid (H)**	11001	55	0.5 (0.4–0.7)	0.2	81	0.7 (0.6–0.9)	0.2	81	0.7 (0.6–0.9)	0.2
**Moxifloxacin (MFX)**	517	50	8.8 (6.6–11.5)	1.0	50	8.8 (6.6–11.5)	1.0	50	8.8 (6.6–11.5)	1.0
**Ofloxacin (OFX)** **/levofloxacin (LFX)**	3809	93	2.4 (1.9–2.9)	0.5	94	2.4 (2.0–2.9)	0.5	94	2.4 (2.0–3.0)	0.5
**Amikacin (AM)**	809	6	0.7 (0.3–1.6)	0.2	6	0.7 (0.3–1.6)	0.2	6	0.7 (0.3–1.6)	0.2
**Kanamycin (KM)**	943	25	2.6 (1.7–3.8)	0.8	25	2.6 (1.7–3.8)	0.8	25	2.6 (1.7–3.8)	0.8
**Capreomycin (CM)**	810	109	**11.9** (9.8–14.1)	3.9	109	**11.9** (9.8–14.1)	3.9	109	**11.9** (9.8–14.1)	3.9
**Streptomycin (S)**	2204	16	0.7 (0.4–1.2)	0.3	16	**0.7** (0.4–1.2)	0.3	16	**0.7** (0.4–1.2)	0.3
**Ethionamide and prothionamide (ETO/PTO)**	298	0	**0.0** (0.0–1.2)	0.0	7	2.3 (0.9–4.7)	1.2	7	2.3 (0.9–4.7)	1.2
**Pyrazinamide (Z)**	2595	59	2.2 (1.7–2.9)	0.8	67	2.5 (2.0–3.2)	0.9	83	3.1 (2.5–3.8)	1.0

The resistance missed corresponds to the false-susceptible isolates divided by the “corrected phenotype”, consisting of the sum of phenotypically resistant isolates and false-susceptible isolates (the probable smallest and largest figures in each category are shown in bold). The difference in sensitivity was calculated by subtracting the sensitivity for the “observed phenotype” from the sensitivity of the “corrected phenotype” (both sensitivities are plotted in figure 2a).

Some genotypic diagnostic tests, such as the Xpert MTB/RIF (Cepheid, Sunnyvale, CA, USA) and Hain line-probe assays (Hain Lifescience, Nehren, Germany), infer the presence of clinically relevant mutations when wild-type assay probes do not anneal to MTBC sequence in a clinical sample. When they do this, they are effectively using an indiscriminate “all mutations” approach of predicting phenotypic resistance. We looked at the potential effect of including all observed mutations in the entire gene on our prediction estimates, rather than just the graded mutation set. The gains in sensitivity compared with the Hi+Mo+Mi set was marked in some cases (mean 11.5%, range 0.9–32.6%), such as ETO/PTO and Z, but also resulted in a large decrease in specificity (mean −10.8%, range −26.5–−1.5%) (figure 2a and b). Notably, these decreases in specificity were probably an underestimate, since synonymous mutations that can cause systematic false-resistance results were excluded from this study [32].

### Assessment of the interpretation guidelines of the Hain GenoType MTBDR*plus* v2.0 and MTBDR*sl* v2.0

Based on the package inserts of the Hain GenoType MTBDR*plus* v2.0, 32 mutations are identified as mutations that confer resistance to isoniazid (H) or rifampicin (R) [33]. 18 of these mutations had either high, moderate or minimal confidence iBCVs, whereas the remaining 14 where either indeterminate, occurred only in combination with other mutations or were not evaluated in this review (online supplementary material 12, table S12.1). Of the 19 genetic markers identified as predictors of resistance to fluoroquinolones, KM, AM or CM, as defined by the package insert for the GenoType MTBDR*sl* v2.0 [34], 10 mutations were found to be indeterminate in our study, were only found in combination with other mutations or were located in a region not considered for at least one antibiotic in this review (online supplementary material 12, table S12.1).

## Discussion

Rapid evidence-based triaging of patients with drug resistant MTBC strains to appropriate drug-resistant TB treatment regimens can only be achieved using genotypic DST methods. Yet, in practice, our understanding of the consequences of classifying patient MTBC strains as “resistant” based on the detection of certain mutations is biased by subjective methods and limited datasets. A case point is the rapid detection of R resistance in clinical MTBC samples. This is now largely achieved using molecular tests, but the emergence of data on discrepancies between genotypic and phenotypic DST, and some systematic false-positive results have created some uncertainty regarding the use of molecular data for early management of patients [35–39]. Using an expert, consensus-driven approach, we developed and verified a standardised procedure to assess the level of confidence in the association between individual mutations and clinically relevant phenotypic drug resistance in MTBC. Our comprehensive approach provides clear, objective and quantitative estimates of the correlation of genotype with phenotypic resistance that is consistent with methods previously established for evidence-based medicine. These findings have immediate implications for molecular and WGS diagnostic assays currently under development, as well as for the interpretation of existing commercially available molecular DST assays. For example, to our knowledge, the *eis* c-2a mutation, which is interpreted as conferring KM resistance in the package insert of the MTBDR*sl* v2.0 assay, has only ever been observed in two KM-resistant strains that also harbour the high-confidence *eis* c-14t mutation (online supplementary material 12) [34, 40]. Consequently, there is currently no convincing evidence that the *eis* c-2a mutation alone is a valid marker for phenotypic KM resistance and the interpretation of the assay should probably be changed to remove this mutation from consideration or require co-occurrence of the *eis* c-14t mutation for clinically relevant interpretation. This example illustrates the potential value of our findings to guide molecular diagnostics developers in terms of which mutations to include and exclude in their assays and interpretation guides, as well as to help regulators evaluating manufacturer claims and clinicians to minimise systematic false-positive and false-negative results [32]. In addition, our results confirmed previous findings that some potentially clinically relevant resistance mutations could be systematically overlooked if certain phenotypic methods are used for DST [35].

Likelihood ratios are not only useful in computing the (post-test) probability of a diagnosis, but can also be used to evaluate the association between a mutation and a given phenotype of interest, in this case drug resistance [20, 41]. Using likelihood ratio thresholds, we classified observed variants as high, moderate or minimal confidence resistance mutations (table 2). While likelihood ratios are commonly used to refine clinical judgements and pretest probabilities, they have not been used previously in this manner for predicting phenotypic TB drug resistance. This approach has two main strengths. First, the likelihood ratio is a universal measure of association in diagnostics that is not affected by local or regional prevalence of drug resistance [42]. Second, unlike sensitivity and specificity, which are often used to assess resistance mutations as predictors of phenotypic resistance, likelihood ratios do not lead to an exaggeration of the benefits of a test or the strength of an association [43], since they simply provide a multiplier for the pretest probability of resistance. In particular, a high sensitivity and a high specificity do not ensure that the positive result of a diagnosis is correct if the underlying condition is exceedingly rare. However, the grading system actually does not take into account the uncertainty (95% confidence intervals) around the likelihood ratio estimate for mutations that are positively associated with resistance. This means that mutations can be graded as high confidence, despite having been observed in only few resistant isolates (*e.g.* the *tlyA* N236K mutation was assigned a high confidence ICV despite occurring in just three resistant and one susceptible isolates; online supplementary material S7). However, the confidence level in the grading of each mutation from this study must necessarily be regarded as provisional, since it could change in either direction as more data are accumulated. This is an inevitable attribute of any evidence-based approach, as any conclusion is open to revision when new evidence comes to light [44].

Applying our grading scheme to a large, systematically collected set of MTBC sequencing and phenotypic data, we were able to identify a total of 286 high, moderate and minimal confidence corrected BCVs (table 3). The resulting diagnostic sensitivities and specificities compared with phenotypic DST can be found in figure 2a and b.

The WHO has defined a specificity of ≥98% and a sensitivity of >95% (for R) or >90% (for H, fluoroquinolones, KM, AM and CM) compared to phenotypic reference standards as a requirement for diagnosing drug-resistant TB [31]. While the diagnostic sensitivities we observed in this study were lower than the WHO thresholds using only the graded mutations to predict resistant phenotypes, this is probably an underestimate of maximum potential sensitivity of genotypic prediction of resistance due to a combination of five important limitations. First, and most fundamental, are the genes and mutations considered. In this systematic review, we were limited to including only those genes and mutations previously documented to be associated with resistance and included in the published literature. While to our knowledge, our review is one of the most comprehensive yet completed, the global knowledge base on all genes associated with resistance is still growing, and we know that certain genes, for example, *ahpC* were not included as potential predictors of H resistance. Additionally, not all studies included data on all known resistance associated genes, which limited the sensitivity. Second, current sequencing technologies have varying capabilities to detect low frequencies (<20%) of resistant strains mixed with susceptible stains relative to phenotypic testing that can detect resistant strains making up only 1% of the total population [45]. This can be a major source of discordance between the detected genotype (apparently wild-type) and a resistant phenotype for some drugs, particularly the fluoroquinolones [9]. Third, breakpoint artefacts (*i.e.* inappropriately high critical concentrations) can be a major source of misclassification of phenotypes. This is well illustrated for CM, for which 11.9% (95% CI 9.8–14.1%) of strains harbouring markers of resistance were missed by phenotypic DST (table 4) [28]. Fourth, the specific biology and genetics of some resistance mechanisms occasionally limited the sensitivity of our method. For resistance caused by loss-of-function mutation in a nonessential gene (*e.g. pncA*), the number of different resistance mutations was very large and, consequently individual mutations were infrequent [11]. Our grading scheme scored such mutations as indeterminate until sufficient evidence can be gathered. Additionally, resistance mutations with MIC distributions that overlap substantially with the MIC distribution of susceptible strains are inherently difficult to distinguish from mutations not associated with resistance. This is because the MIC distributions of these mutations are truncated by the CC, which means that a mutant strain will not consistently test resistant due to the inherent variation in phenotypic testing. This phenomenon was most noticeable for the *eis* g-37t and c-12t mutations, which reduced confidence in the association and did not meet statistical significance for an association with KM resistance after the more conservative p-value correction was applied (table 3) [46]. Fifth, synonymous mutations were excluded from this analysis because these are not reported routinely in association studies. However, it is known that these mutations can sometimes confer resistance [47, 48].

The specificities of the corrected iBCVs were usually superior to the sensitivities and would be 100% if an expert rule was adopted for the genotype to overrule the phenotype whenever a high, moderate or minimal confidence mutation was detected (figure 2a and b). This is especially relevant for mutations affected by breakpoint artefacts, mutations that confer modest MIC increases, such as the “disputed” *rpoB* mutations [49], and drugs for which resistance is currently defined inconsistently on the phenotypic level. The latter point is best illustrated with the *gyrA* A90V mutation, which confers low-level resistance to MFX [50]. Consequently, it has a high specificity as predictor of phenotypic resistance when the phenotypic standard is the Clinical and Laboratory Standards Institute critical concentration of 0.5 mg·L^−1^ with 7H10, but not when the WHO critical concentration of 2 mg·L^−1^ on 7H10 medium is used as the reference [51]. Until critical concentrations are harmonised (which is particularly important for fluoroquinolones, where the evidence is mounting that strains with slightly elevated MICs might still be treatable [52]), and genotypic interpretations adjusted accordingly, this mutation and related mutations will continue to pose diagnostic challenges.

Additional improvements to the graded mutation list could be implemented based on lessons learned from limitations of our study. Our study was limited by the fact that it mostly relied on amplicon-based sequencing data, which meant that the underlying population structure could not be taken into account. If WGS data had been available for all strains, resistance mutations that are currently classified as indeterminate or potentially even as not associated with resistance because they only confer modest MIC increases, as was the case for *eis* g-37t and c-12t, could be identified as resistance associated by the virtue of them being homoplastic (*i.e.* arising in unrelated isolates independently [26]). Such observations could help focus future MIC testing and/or allelic exchange experiments to clarify their MIC ranges and confirm or refute an association with resistance [46]. Conversely, mutations that are not homoplastic, such as *gidB* E92D, that are known lineage markers for particular genotypes and not markers for resistance could be excluded [26]. However, even using WGS, some manual curation based on an assessment of the mode of action of the antibiotic may still be required to remove spurious associations, as was the case for the *rrs* a1401g mutation.

The methods presented in this study will be used as a standardised analytical approach for assessing potential resistance mutations in the Relational Sequencing TB Data Sharing Platform currently available at https://platform.reseqtb.org/. The ReSeqTB platform serves as a globally harmonised knowledge base for the curation, validation and interpretation of existing and newly created genotypic and phenotypic data for TB drug resistance correlations [18]. In this context, the grading system presented here will be refined further by taking the following criteria into consideration: phylogenetic information, laboratory evidence (*e.g.* MIC values, epidemiological cut-offs and/or pharmacokinetics/pharmacodynamics-driven thresholds, biochemical assays and site-directed mutagenesis) and clinical evidence.

This study establishes the first confidence-graded list of mutations for predicting drug resistance, and as such should serve as a gene target guide for developing new molecular diagnostics, and as a tool for supporting the clinical interpretation of existing molecular diagnostics such as the Hain GenoType assays. Once incorporated into the ReSeqTB knowledge base, we expect the confidence-graded list of mutations to improve in precision iteratively as data are accumulated, and it will be revised annually through an expert review process similar to the methods established for the Stanford HIV Drug Resistance Database (https://hivdb.stanford.edu). This will be of particular value for the interpretation of WGS-based *in vitro* diagnostics that are currently being piloted as decision support tools for rapid and comprehensive characterisation of clinically relevant resistance to guide individualised treatment regimens containing the most effective, least toxic drug combinations. Ultimately, we aim to provide a comprehensive and user friendly tool to assist clinicians with the interpretation of resistance mutations in MTBC.

## Supplementary material

10.1183/13993003.01354-2017.Supp1 **Please note:** supplementary material is not edited by the Editorial Office, and is uploaded as it has been supplied by the author.
Supplementary materials 1, 4, 5, 6 and 11 ERJ-01354-2017_Suppl_1-4-5-6-11
Supplementary materials 2, 3, 7, 8, 9, 10 and 12 ERJ-01354-2017_Suppl_2-3-7-8-9-10-12

## Disclosures

10.1183/13993003.01354-2017.Supp2 D. Alland ERJ-01354-2017_Alland
I. Comas ERJ-01354-2017_Comas
C.M. Denkinger ERJ-01354-2017_Denkinger
K. Dheda ERJ-01354-2017_Dheda
D. Hanna ERJ-01354-2017_Hanna
C.U. Koser ERJ-01354-2017_Koser
C. Lange ERJ-01354-2017_Lange
R. Liwski ERJ-01354-2017_Liwski
S. Niemann ERJ-01354-2017_Niemann
L. Rigouts ERJ-01354-2017_Rigouts
M. Schito ERJ-01354-2017_Schito
