# Supplementary material for: A standardised method for interpreting the association between mutations and phenotypic drug resistance in Mycobacterium tuberculosis
Source: Eur Respir J. 2017 Dec 28;50(6):1701354. doi: 10.1183/13993003.01354-2017 (PMC5898944; doi:10.1183/13993003.01354-2017)
Supplement: Supplementary file 1 [file ERJ-01354-2017_Suppl_1-4-5-6-11.pdf]

A standardised method for interpreting the association between mutations and phenotypic drug-resistance in *Mycobacterium tuberculosis*

Authors:

Paolo Miotto, Belay Tessema, Elisa Tagliani, Leonid Chindelevitch, Angela M. Starks, Claudia Emerson, Debra Hanna, Peter S. Kim, Richard Liwski, Matteo Zignol, Christopher Gilpin, Stefan Niemann, Claudia M. Denkinger, Joy Fleming, Rob Warren, Derrick Crook, James Posey, Sebastien Gagneux, Sven Hoffner, Camilla Rodrigues, Iñaki Comas, David M. Engelthaler, Megan Murray, David Alland, Leen Rigouts, Christoph Lange, Keertan Dheda, Rumina Hasan, Uma Devi K. Ranganathan, Ruth McNerney, Matthew Ezewudo, Daniela M. Cirillo, Marco Schito, Claudio U. Köser, Timothy C. Rodwell.

Supplementary 1

Table S1.1. Loci of interest considered for retrieving mutations associated with drug resistance. References reported in the table have been chosen to justify the selection of the loci for each drug.

Drug	Gene name	Gene locus	References
Amikacin (AM)	*rrs*	*MTB000019*	Maus 2005 [1,2]; Reeves 2015 [3]
Capreomycin (CM)	*rrs*	*MTB000019*	Maus 2005 [1,2]; Reeves 2015 [3]
	tlyA	*Rv1694*	Maus 2005 [1,2]; Johansen 2006 [4]
Kanamycin (KM)			
	rrs	*MTB000019*	Maus 2005 [1,2]; Reeves 2015 [3]
	eis	*Rv2416c*	Zaunbrecher 2009 [7]
	whiB7	*Rv3197A*	Köser 2013 [5]; Reeves 2013 [6]
Streptomycin (S)	*rpsL*	*Rv0682*	Finken 1993 [8]
	tap	*Rv1258c*	Köser 2013 [5]; Reeves 2013 [6]
	rrs	*MTB000019*	Finken 1993 [8]
	whiB7	*Rv3197A*	Köser 2013 [5]; Reeves 2013 [6]
	gidB	*Rv3919c*	Okamoto 2007 [9]; Wong 2011 [10]
Levofloxacin (LFX)	*gyrA*	*Rv0006*	Takiff 1994 [11]; Nebenzahl-Guimaraes 2014 [12]
	gyrB	*Rv0005*	Kocagöz 1996 [13]; Nebenzahl-Guimaraes 2014 [12]
Ofloxacin (OFX)	*gyrA*	*Rv0006*	Takiff 1994 [11]; Nebenzahl-Guimaraes 2014 [12]
	gyrB	*Rv0005*	Kocagöz 1996 [13]; Nebenzahl-Guimaraes 2014 [12]
Moxifloxacin (MFX)	*gyrA*	*Rv0006*	Takiff 1994 [11]; Nebenzahl-Guimaraes 2014 [12]
	gyrB	*Rv0005*	Kocagöz 1996 [13]; Nebenzahl-Guimaraes 2014 [12]
Isoniazid (H)	*mshA*	*Rv0486*	Vilchèze 2008 [14]; Vilchèze 2011 [15]
	mabA (fabG1)	*Rv1483*	Ando 2014 [16]
	inhA	*Rv1484*	Banerjee 1994 [17]; Kapur 1995 [18]; Nebenzahl-Guimaraes 2014 [12]
	katG	*Rv1908c*	Zhang 1992 [19]; Ando 2011 [20]; Nebenzahl-Guimaraes 2014 [12]
	furA	*Rv1909c*	Ando 2011 [20]
Ethionamide (ETO)	*mshA*	*Rv0846*	Vilchèze 2008 [14]; Vilchèze 2011 [15]
	mabA (fabG1)	*Rv1483*	Ando 2014 [16]
	inhA	*Rv1484*	Banerjee 1994 [17]; Nebenzahl-Guimaraes 2014 [12]
	ethA	*Rv3199c*	DeBarber 2000 [21]; Dover 2007 [22]; Gopal 2015 [23]
Prothionamide (PTO)	*mshA*	*Rv0846*	Vilchèze 2008 [14]; Vilchèze 2011 [15]
	mabA (fabG1)	*Rv1483*	Ando 2014 [16]
	inhA	*Rv1484*	Banerjee 1994 [17]; Nebenzahl-Guimaraes 2014 [12]
	ethA	*Rv3199c*	DeBarber 2000 [21]; Dover 2007 [22]; Gopal 2015 [23]
Pyrazinamide (Z)	*pncA*	*Rv2043c*	Scorpio 1996 [24]
Rifampicin (R)	*rpoB*	*Rv0667*	Telenti 1993 [25]; Williams 1998 [26]; Schön 2013 [27]

References

1. Maus CE, Plikaytis BB, Shinnick TM. Molecular analysis of cross-resistance to capreomycin, kanamycin, amikacin, and viomycin in *Mycobacterium tuberculosis*. *Antimicrob Agents Chemother* 2005; **49**(8): 3192-7.
2. Maus CE, Plikaytis BB, Shinnick TM. Mutation of *tlyA* confers capreomycin resistance in M*ycobacterium tuberculosis*. *Antimicrob Agents Chemother* 2005; **49**(2): 571-7.
3. Reeves AZ, Campbell PJ, Willby MJ, Posey JE. Disparities in capreomycin resistance levels associated with the *rrs* A1401G mutation in clinical isolates of *Mycobacterium tuberculosis*. *Antimicrob Agents Chemother* 2015; **59**(1): 444-9.
4. Johansen SK, Maus CE, Plikaytis BB, Douthwaite S. Capreomycin binds across the ribosomal subunit interface using tlyA-encoded 2'-O-methylations in 16S and 23S rRNAs. *Mol Cell* 2006; **23**(2): 173-82.
5. Köser CU, Bryant JM, Parkhill J, Peacock SJ. Consequences of *whiB7* (Rv3197A) mutations in Beijing genotype isolates of the *Mycobacterium tuberculosis* complex. *Antimicrob Agents Chemother* 2013; **57**(7): 3461.
6. Reeves AZ, Campbell PJ, Sultana R, et al. Aminoglycoside cross-resistance in *Mycobacterium tuberculosis* due to mutations in the 5' untranslated region of *whiB7*. *Antimicrob Agents Chemother* 2013; **57**(4): 1857-65.
7. Zaunbrecher MA, Sikes RD, Metchock B, Shinnick TM, Posey JE. Overexpression of the chromosomally encoded aminoglycoside acetyltransferase eis confers kanamycin resistance in *Mycobacterium tuberculosis*. *Proc Natl Acad Sci U S A* 2009; **106**(47): 20004-9.
8. Finken M, Kirschner P, Meier A, Wrede A, Böttger EC. Molecular basis of streptomycin resistance in *Mycobacterium tuberculosis*: alterations of the ribosomal protein S12 gene and point mutations within a functional 16S ribosomal RNA pseudoknot. *Mol Microbiol* 1993; **9**(6): 1239-46.

9. Okamoto S, Tamaru A, Nakajima C, et al. Loss of a conserved 7-methylguanosine modification in 16S rRNA confers low-level streptomycin resistance in bacteria. *Mol Microbiol* 2007; **63**(4): 1096-106.

10. Wong SY, Lee JS, Kwak HK, Via LE, Boshoff HI, Barry CE. Mutations in *gidB* confer low-level streptomycin resistance in *Mycobacterium tuberculosis. Antimicrob Agents Chemother* 2011; **55**(6): 2515-22.

11. Takiff HE, Salazar L, Guerrero C, et al. Cloning and nucleotide sequence of *Mycobacterium tuberculosis gyrA* and *gyrB* genes and detection of quinolone resistance mutations. *Antimicrob Agents Chemother* 1994; **38**(4): 773-80.

12. Nebenzahl-Guimaraes H, Jacobson KR, Farhat MR, Murray MB. Systematic review of allelic exchange experiments aimed at identifying mutations that confer drug resistance in *Mycobacterium tuberculosis*. *J Antimicrob Chemother* 2014; **69**(2): 331-42.

13. Kocagöz T, Hackbarth CJ, Unsal I, Rosenberg EY, Nikaido H, Chambers HF. Gyrase mutations in laboratory-selected, fluoroquinolone-resistant mutants of *Mycobacterium tuberculosis* H37Ra. *Antimicrob Agents Chemother* 1996; **40**(8): 1768-74.

14. Vilchèze C, Av-Gay Y, Attarian R, et al. Mycothiol biosynthesis is essential for ethionamide susceptibility in *Mycobacterium tuberculosis*. *Mol Microbiol* 2008; **69**(5): 1316-29.

15. Vilchèze C, Av-Gay Y, Barnes SW, et al. Coresistance to isoniazid and ethionamide maps to mycothiol biosynthetic genes in *Mycobacterium bovis*. *Antimicrob Agents Chemother* 2011; **55**(9): 4422-3.

16. Ando H, Miyoshi-Akiyama T, Watanabe S, Kirikae T. A silent mutation in *mabA* confers isoniazid resistance on *Mycobacterium tuberculosis*. *Mol Microbiol* 2014; **91**(3): 538-47.

17. Banerjee A, Dubnau E, Quemard A, et al. inhA, a gene encoding a target for isoniazid and ethionamide in *Mycobacterium tuberculosis*. *Science* 1994; **263**(5144): 227-30.

18. Kapur V, Li LL, Hamrick MR, et al. Rapid Mycobacterium species assignment and unambiguous identification of mutations associated with antimicrobial resistance in *Mycobacterium tuberculosis* by automated DNA sequencing. *Arch Pathol Lab Med* 1995; **119**(2): 131-8.

19. Zhang Y, Heym B, Allen B, Young D, Cole S. The catalase-peroxidase gene and isoniazid resistance of *Mycobacterium tuberculosis*. *Nature* 1992; **358**(6387): 591-3.

20. Ando H, Kitao T, Miyoshi-Akiyama T, Kato S, Mori T, Kirikae T. Downregulation of *katG* expression is associated with isoniazid resistance in *Mycobacterium tuberculosis*. *Mol Microbiol* 2011; **79**(6): 1615-28.

21. DeBarber AE, Mdluli K, Bosman M, Bekker LG, Barry CE. Ethionamide activation and sensitivity in multidrug-resistant *Mycobacterium tuberculosis*. *Proc Natl Acad Sci U S A* 2000; **97**(17): 9677-82.

22. Dover LG, Alahari A, Gratraud P, et al. EthA, a common activator of thiocarbamide-containing drugs acting on different mycobacterial targets. *Antimicrob Agents Chemother* 2007; **51**(3): 1055-63.

23. Gopal P, Dick T. The new tuberculosis drug Perchlozone® shows cross-resistance with thiacetazone. *Int J Antimicrob Agents* 2015; **45**(4): 430-3.

24. Scorpio A, Zhang Y. Mutations in *pncA*, a gene encoding pyrazinamidase/nicotinamidase, cause resistance to the antituberculous drug pyrazinamide in tubercle bacillus. *Nat Med* 1996; **2**(6): 662-7.

25. Telenti A, Imboden P, Marchesi F, et al. Detection of rifampicin-resistance mutations in *Mycobacterium tuberculosis*. *Lancet* 1993; **341**(8846): 647-50.

26. Williams DL, Spring L, Collins L, et al. Contribution of *rpoB* mutations to development of rifamycin cross-resistance in *Mycobacterium tuberculosis*. *Antimicrob Agents Chemother* 1998; **42**(7): 1853-7.

27. Schon T, Jureen P, Chryssanthou E, et al. Rifampicin-resistant and rifabutin-susceptible *Mycobacterium tuberculosis* strains: a breakpoint artefact? *J Antimicrob Chemother* 2013; **68**(9): 2074-7.

Supplementary 4

Assessment Methods and utilization of data

**Main possible sources of bias**	**Bias mitigation**
Screening<br>- Papers not accessible have been excluded	Screening<br>- High number of papers considered should compensate for the papers excluded *a priori*
Sample selection<br>- Sampling strategy has not been systematically tracked<br>- Due to their high number, studies providing sequencing only of drug resistant cases have been included	Sample selection<br>- Studies providing DST only for mutated strains have been excluded<br>- Studies providing sequencing data for discrepancies or for a subset of data selected as representative of the entire data set (e.g. % of susceptible cases or random selected subgroup and similar) have been excluded
Data sets<br>- Unless clearly specified in the study, reporting of multiple mutations was considered based on the data reported in the tables<br>- In almost all the cases, double mutations reported in the studies are not clearly classified as "mixed infection" or "real presence of multiple mutations on the same genome"<br>- Hetero-resistance defined as the presence of wild-type + mutated alleles has not been considered and only the mutated allele has been reported	Data sets<br>- Only data produced for the published study have been considered in order to avoid data duplication (e.g. data reported as produced in references have not been considered)<br>- Whenever identifiable, replicated data sets have been excluded

Country representativeness was considered as "indicative" because (i) origin of samples is not always clearly stated and/or (ii) origin of samples is often referred to by regions (e.g. Europe, North America) rather than by specific countries.

The quality of included studies was appraised with a modified Quality Assessment of Diagnostic Accuracy Studies (QUADAS-2) tool. QUADAS-2 consists of four domains: patient selection, index test, reference standard, and flow and timing. Risk of bias was determined.

Domain 1: Sample Selection

Studies included in the systematic review considered culture isolates which probably introduced bias. Most published data is focused on drug resistant and/or MDR–TB patients and only a relatively small number of studies consider appropriate proportions of susceptible cases in order to provide statistical confidence to assessments made correlating genotype to phenotype. Thus, the predominant selection strategy was by convenience or based on a case-control design, representing a bias source. Including surveillance data could provide a mitigation strategy.

Time and geographical regions could potentially represent a source of bias in terms of circulating strains (*e.g.* samples over-representing specific geographical settings and/or specific timeframes). However, timing and geographical regions of isolation has been tracked.

Cases not matching the review question are likely to be successfully excluded: studies providing partial data sets (*e.g.* sequencing/phenotypic data for discrepancies or for data subsets) have been excluded.

Domain 2: Index Test

Different index tests have been used. Despite the main purpose of the review, to provide a correlation between a given mutation observed and it's observed phenotypic drug susceptibility results, rather than evaluate the performance of a test/technology in detecting the mutations, the possibility to have studies reporting errors during the analysis of NGS results could not be excluded.

Considering the sample selection strategy, most of the cases have been analysed in an un-blinded manner. This could affect the results because the operator could pay more attention in identifying mutations in resistant cases (*e.g.* looking for smaller peaks in the electropherogram or lower percentage of nucleotides in NGS data) or considering clean wild-type sequences in susceptible cases (*e.g.* overlooking double signals).

In addition, assessment of reporting of multiple mutations could be biased due to the performance limits of the sequencing chemistry and the technology utilized (*e.g.* inability to detect and discriminate between mixed populations and real double mutations) as well as the data reporting system (*e.g.* most of the studies reporting mutations known or assumed to be involved in drug resistance and do not report any additional mutations or mutations known or assumed to be not involved in drug resistance including silent mutations or mutations known to be lineage-specific. Also most of the studies relied on targeted sequencing, thus mutations outside the targeted regions are not detected); therefore the data presented is not truly indicative of the true genomic sequence).

Domain 3: Reference Standard
If the phenotypic reference standard used was WHO-endorsed and performed as per WHO recommendations, it was judged as 'low risk'; if the phenotypic reference standard was performed by other methods or procedures, it was judged to be at 'high risk' for inaccuracies.

Due to the well-known limits of the culture-based PZA testing methods, for PZA resistance the enzymatic-based assay (Wayne's method) was also used as alternative reference test.

Domain 4: Flow and Timing (time frame)
The interval between when the sequencing and phenotypic reference standard was done is not considered a source of bias for this study.

Different reference standards were used in the different studies. Stratifying the data by reference standard methods used (liquid/solid culture, absolute/proportion method, critical concentration/minimum inhibitory concentration) should minimize biases introduced by the technique used for phenotypic DST testing. Quality of reference standard results could not be easily and objectively assessed; however, whenever possible and clearly stated in the study, participation of reference laboratories with external quality assurance programs was noted. In addition, we considered only studies providing phenotypic testing and sequencing for all of the tested samples, thus minimizing potential biases introduced by the inclusion of partial data sets.

Supplementary 5

Development of a standardized protocol for the assessment of resistance-associated mutations

Traditionally, likelihood ratios (LRs) are a metric measuring the strength of association between the outcome of a test and a diagnosis, and are often used to guide clinical decision-making. In this manuscript we used them as a measure of the strength of association between the presence of a mutation and the drug resistance phenotype. Under the null hypothesis of no association, the LR is expected to be 1, but deviations from this value can be due to both a true association as well as a variety of factors such as sampling effects. Generally speaking, the higher the likelihood ratio, the more the probability of the outcome of interest (here, drug resistance) increases after a positive test or observation (here, detection of the mutation). However, the baseline probabilities must not be ignored, even a mutation with a high LR is not conclusive proof of drug resistance in settings whre the prior (or pre-test) probability of drug resistance is low. In practice of evidence-based medicine, it is generally assumed that LRs from 2 to 5 yield small increases in the post-test probability, whereas ratios 5 to 10 denote moderate increases, and LRs above 10 indicate large increases. For ratios less than unity, the post-test probability is in fact lower than the pre-test probability, and the smaller the LR, the lower the post-test probability of the outcome of interest.
Data from the literature were used to calculate the frequency of each mutation in resistant (DR) and susceptible (DS) MTBC isolates and then used to derive a LR, which, in this case, represented the probability of observing a given mutation in a phenotypically resistant isolate divided by the probability of observing the mutation in a phenotypically susceptible isolate:

$$LR+= \frac{\Pr(M+|DR+)}{\Pr(M+|DR-)} \quad \text{or equivalently} \quad LR+= \frac{sensitivity}{1-specificity}$$

In addition, odds ratios (OR), another metric commonly adopted in genome-wide association studies, were used to evaluate the association of the systematically reviewed genotypic and phenotypic data. The OR is the ratio of the odds of an event ("the presence of a mutation") occurring in one group ("phenotypically drug resistant isolates") to the odds of it occurring in another group ("phenotypically drug susceptible isolates"). It is a measure of effect size, describing the strength of association between two binary values, and is defined as:

$$OR = \frac{\Pr(M+|DR+)}{\Pr(M+|DR-)}\ \frac{\Pr(M-|DR-)}{\Pr(M-|DR+)} \quad \text{or equivalently} \quad OR = \frac{sensitivity \times specificity}{(1-sensitivity)\times(1-specificity)}$$

In the above formulas, "M+" and "M-" denote the presence and absence of a mutation, respectively, whereas "DR+" and "DR−" denote a resistant phenotype and a susceptible phenotype, respectively.
The interpretation of OR is similar to the interpretation of the LR, and can be summarized as follows:

OR=1 the presence of a mutation does not affect the odds of phenotypic resistance
OR>1 the presence of a mutation associated with higher odds of phenotypic resistance
OR<1 the presence of a mutation associated with lower odds of phenotypic resistance

In our approach, LRs were used for objectively evaluating whether mutations were positively associated with phenotypic resistance. Using this rationale, the thresholds that are most commonly used in evidence-based medicine[1,2,3] to grade MTBC mutations (Figure 1) were adopted.

In addition, *p*-values associated with the respective LR and OR scores were considered; the *p*-value can be interpreted as the probability of occurrence of a result as the one observed if the null hypothesis of no association is true. For the purposes of this evaluation, a *p*-value below 0.05 was designated as the threshold for indicating a nominally statistically significant difference between drug resistant and drug susceptible groups with respect to the presence of a mutation, whereas a *p*-value above 0.05 indicates that there is no evidence of a statistically significant difference between the groups. If the *p*-value associated with the LR and the OR was not significant (above 0.05), the association between the mutation and the phenotype was considered to be "indeterminate". The calculated *p*-values provide the putative significance level of the association between the presence of a mutation and drug resistance. These nominal *p*-values (also referred as "uncorrected *p*-values" in the manuscript and related Supplementary material) may, however, be misleading given that multiple mutations are simultaneously evaluated for association with the drug resistance phenotype. Even under the null hypothesis of no association, when the *p*-values are uniformly distributed in the interval (0, 1), on average one in every 20 association tests will appear to be significant. From a clinical point of view, this means that some of the mutations will be incorrectly pointing towards drug resistance when they should actually not. For this reason we adopted a correction procedure known as the FDR (false discovery rate), which effectively confers statistical significance only to those associations that pass a more stringent threshold, adjusted based on the number of tests performed. The FDR-corrected *p*-values were used as the basis for the final significance level of association between the presence of a mutation and drug resistance. However, not all tests were counted for the purposes of this correction, as described below in "criteria used to classify genetic variants for the correction of the *p*-value".

Nonsense mutations (i.e. premature stop codons) and frameshift mutations in non-essential resistance genes can be predicted with confidence to cause a loss-of-function phenotype[4,5,6,7]. Therefore, in addition to analyzing these changes individually, these variants were pooled and treated as one class of changes. This rule was only applied to isolates in which indels or nonsense mutations occurred on their own (i.e. isolates with indels or nonsense mutations that coincided with further changes were not considered for this calculation). By contrast, in-frame indels in essential genes were only analyzed individually given that it was not possible to predict the effect of such variants *a priori*. Unless otherwise stated, indels are frameshift (whereas inframe mutations have been explicitly specified).

Essential and non-essential genes were defined according to the information reported in the TubercuList database[8], available at http://tuberculist.epfl.ch/ (version 2.6, Release 27. Accessed Nov 18th, 2016). Silent mutations (i.e. nucleotide changes that do not alter the amino acid sequence of the protein) were tracked but considered as wild-type in the analysis. For this reason, variants found to be in association with silent mutations were merged if also found not in combination with silent mutations. (e.g. the entries "S531L" and "S531L+G536G" were combined to form "S531L (merged entry)").

The synonymous mutations that were reported in the included studies can be found in the following table and were considered as wild-type throughout the analysis.

**Gene**	**Synonymous mutations**
eis	S48S, P90P
gidB	A205A
gyrA	A125A, L197L, L198L, T272T, A343A, A384A, E485E, L549L, I568I, A581A, V678V, G809G
gyrB	T625T
inhA	G3G, L44L, L88L, S142S, G205G
mabA	L203L, G241G
katG	D33D, E99E, A110A, G205G, E261E, K239K, P241P, A281A, A291A, T308T, T326T, K327K, T344T, L611L
mshA	G106G, D208D, A298A, C409C, L244L, L261L
pncA	F13F, A20A, A26A, L27L, A36A, A38A, A39A, A46A, G55G, K96K, S65S, S74S, G75G, T76T, G78G, G124G, D129D, C138C, G150G, E173E
rpoB	D184D, F514F, L521L, T525T, R529R, L533L, G534G, G536G, S539S, R542R, G544G, R548R, V550V, P552P, E562E, G566G, S576S, P657P, L859L, G957G, A1156A
rpsL	R12R, S17S, A23A, L24L, R30R, G32G, C34C, T39T, T41T, P45P, S47S, A48A, V52V, L57L, S59S, V61V, V63V, T64T, A65A, I67I, G71G, S78S, L81L, R83R, G84G, P91P, R94R, K121K
tlyA	L11L

Criteria used to classify genetic variants for the correction of the *p*-value

The following criteria were used to classify genetic variants for the correction of the p-value:

1. *to be included:* single variant to be considered for the correction
2. *to be excluded - variant already considered in merged entry for silent mutation:* variant observed in combination with silent mutations, thus a merged entry has been already considered for the correction
3. *to be excluded – other*: genetic variants showing uncertainties and/or not defined (rationale: we cannot use an undefined mutation as a standard variant)
4. *to be excluded - multiple mutations*: genetic variants observed in combination with other variants (rationale: these entries are at risk of bias as reported in our QUADAS analysis)
5. *to be excluded - WT or silent:* WT or entries showing only silent mutations (rationale: these entries do not represent variants. Silent mutations are at risk of bias as reported in our QUADAS analysis and have been considered as wild-type throughout the analysis)
6. *to be excluded – duplicated*: entries highlighted with a "(b)" in the "Mutation" column as described in the following

paragraph (rationale: entries at risk of bias).
7. *considered as pooled*: as previously mentioned, frameshift mutations and premature stop codon were only considered as pooled entries

The nominal *p*-value was used to identify mutations likely to be associated with drug resistance (putative Individual Confidence Values), whereas the corrected *p*-value was used for the final classification (Final Individual Confidence Values).

Data analysis

The following types of studies were included if sequencing and phenotypic DST data were available: randomized controlled trials, cohort studies, case-control studies, cross-sectional studies, case reports, animal studies, and *in vitro* research.
Types of specimens included: clinical MTBC isolates (i.e. *in vitro* mutants were excluded).
For sequencing data, the following definitions were considered:
- wild-type: wild-type refers to a strain, gene, or genetic characteristic that prevails among individuals in natural conditions, as distinct from an atypical or mutant type;
- mutant: mutant refers to any heritable change in the nucleotide sequence in a locus, gene, or genome;
- genetic patterns: combination of different mutations either within the same gene or in different genes.
Index testing for mutation detection: MTBC genomic mutations included in this review were detected and characterized by comparing MTBC nucleotide sequence data with a wild-type, standard reference sequence. Although a variety of sequencing technologies exist, Sanger sequencing, pyrosequencing, and next generation sequencing technologies were the most commonly utilized methods considered in this systematic review *M. tuberculosis* H37Rv (accession number AL123456.2) was used as the reference for the sequence and annotation in this review, with the exception of rifampicin and fluoroquinolones, a different numbering system for relevant codons was used. For rifampicin (R), the *E. coli* codon numbering system was employed[9,10] with the exception of 3 mutations outside the RRDR and reported using the MTB numbering system (H323Y, P206R, and Y314C), whereas for fluoroquinolones (moxifloxacin – MFX, ofloxacin – OFX, levofloxacin – LFX) we used the gyrase B numbering system proposed by Maruri *et al*[11]:

Drug	Gene	Region	*M. tuberculosis* numbering system	Reference numbering system used	Reference
R	*rpoB*	N-term	Cod. 167-172	Cod. 143-148	Campbell 2001 [10]
R	*rpoB*	Cluster I	Cod. 424-456	Cod. 505-537	Campbell 2001 [10]
R	*rpoB*	Cluster II	Cod. 481-494	Cod. 562-575	Campbell 2001 [10]
R	*rpoB*	Cluster III	Cod. 604-610	Cod. 684-610	Campbell 2001 [10]
MFX/OFX/LFX	*gyrB*	QRDR	Cod. 461-499	Cod. 461-499	Maruri 2012 [11]

The reference test for the detection of phenotypic resistance was culture-based phenotypic drug susceptibility testing (refer to Supplementary Material – Data Gathering Form for details). Accordingly, we considered 3 different datasets:
- WHO-endorsed liquid phenotypic drug susceptibility testing
- WHO-endorsed solid phenotypic drug susceptibility testing
- combined phenotypic drug susceptibility testing (derived by combining liquid + solid + WHO-endorsed not specified testing method).

Given the aforementioned limitations of the phenotypic drug susceptibility testing method, we used the following rules for the combined dataset for Z:

Liquid DST	Wayne	Combined
R	R	R
n.a.	R or S	Wayne result
R or S	n.a.	Liquid DST result
S	S	S
S	R	R according to Wayne because of the limitations of liquid DST
R	S	S according to Wayne because liquid DST is more prone to false positives

The amino acid substitution S100F in the *gidB* gene was not considered as a SNP because it represents an error in the earlier H37Rv reference sequences[12]. Therefore, all isolates with this mutation were considered to be wild-type. Some studies reported mutations that fell just outside of the regions that were sequenced based on the methods sections of the studies in question (e.g. outside of the aforementioned *rpoB* clusters). The mutations were highlighted with a '(b)' in the 'Mutation' column in Supplementary S7.1. They were analysed separately given that these could have been genuine mutations because sequencing primers are usually not immediately adjacent to the targeted regions (the primer sequences were not provided to confirm this).

The sensitivity and specificity of predicting phenotypic ofloxacin (OFX) and levofloxacin (LFX) resistance by sequencing were found to be independent of the phenotypic method used, whereas there were substantial differences in specificity for moxifloxacin (MFX) depending on whether liquid or solid DST was used as the reference method (data not shown). Results for OFX and LFX from both testing methods were therefore pooled whereas MFX results were analysed separately for each DST method. To maximise the number of isolates studied and thus increase statistical power, results for ethionamide (ETO) and prothionamide (PTO) were pooled.

The frequency of each mutation in DR and DS cases was determined and stratified according to i) the phenotypic testing method used as the reference (liquid or solid conventional DST) and ii) the geographical origin of the isolates in question. The denominator to calculate these frequencies could differ for different positions in the same gene, as illustrated in the following example:
– Study A reports sequencing results for 100 clinical isolates; target sequence: rifampicin resistance-determining region (RRDR: codons 505-537 of *rpoB*)
– Study B reports sequencing results for the entire *rpoB* gene for 50 clinical isolates
For the mutations in RRDR, the denominator would be 150 (because both the studies A and B are interrogated this regions), whereas the denominator for codons outside this range (i.e. codon <505 and >537) would be 50 (because only study B covered these positions).

Statistical analyses were done using the R statistical computing language[13]. Sensitivity, specificity, positive, negative predictive values, *p*-value estimates, and 95% confidence intervals for each mutation and associated phenotype were calculated using the binomial test[14] (null hypothesis: proportion = ½). Confidence intervals and *p*-values for the odds ratio (OR were calculated using the Fisher exact test[15] (null hypothesis: odds ratio = 1), whereas for the LR, they were calculated using the melded binomial test[16] (null hypothesis: likelihood ratio = 1). Performance characteristics (sensitivity, specificity, and diagnostic accuracy) of different categories of mutations were calculated using the same R script.
The number of isolates with multiple and silent mutations was likely underreported and this estimate should be considered as a risk of potential bias (refer to SUPPL4: QUADAS-2, Domain 2 risk assessment). A comprehensive analysis of multiple mutations could not be provided due the limitation of the data sets analyzed (refer to SUPPL4: QUADAS-2, Domain 2 risk assessment).

Applying the grading system to special cases

– For antimycobacterial drugs with multiple resistance genes "individual confidence value" (ICV) "indeterminate" values were over-ruled by the highest confidence score available. In example 1, the ICVs, for which *rrs* alone was sequenced, overruled the ICVs for both *rrs* and *tlyA*, the latter showing an "indeterminate" confidence value.
– For antimycobacterial drugs with multiple resistance genes "individual confidence value" (ICV) were prioritized for which the greatest number of resistance genes were sequenced (unless the ICVs were "indeterminate" as mentioned above). In examples 2-4, the ICVs, for which both *katG* and *inhA* were sequenced, overruled the ICVs for *inhA* alone, despite the fact that the latter had higher confidence values. The resulting "medium confidence values" (MCV) were calculated for each medium (i.e. liquid, solid, and composite). For antimycobacterial drugs with only a single resistance gene, ICV were by definition equal to the MCV.
– Where the ICV or MCV differed between media the highest confidence value was chosen as the "best confidence value" (BCV) (see cases 24 & 5). If data were only available for a single medium, the corresponding ICV or MCV was by definition equal to the BCV.
– Having assessed variants singly or in combination using ICV, MCV, and BCV, we also calculated equivalent "interpretative" values to extrapolate the data of single mutations to isolates with multiple variants (not considered for the correction of the p-value, and thus not provided with an ICV). A variant was assumed to be necessary and sufficient to cause resistance on its own. So for mutations that harboured multiple variants, the highest confidence value of the individual mutations replaced the confidence level of the combined mutations. This was done for individual media (i.e. by comparing ICVs for antimycobacterial drugs with only a single resistance gene, as shown in case 6, and by comparing MCVs for drugs with multiple resistance gens, as shown in case 7) to yield an "interpretative ICV" (iICV) and "interpretative MCV" (iMCV). Moreover, equivalent "interpretative BCVs" were generated using the BCV for an

overall assessment (case 8; please note that in this case the ICV corresponded to the MCV because a single resistance gene was considered). For isolates with single variants, confidence values were by definition identical to interpretative values (case 8). Indels and nonsense mutations for non-essential genes were considered as pooled for the p-value correction and represent a special case. Here the pooled confidence value overruled the value for an indel/nonsense mutation that occurred either alone or in combination (case 9), provided that pooled confidence was higher. The "interpretative BCVs" (iBCV), which excluded the non-causative mutations from Table 3, was used to calculate the sensitivities and specificities of the mutations (e.g. in Figure 2).

Example cases:

Case	Drug	Mutation	Medium	ICV	MCV
1	CM	rrs c1402t + tlyA n.a.	Composite	[green]	[green]
	CM	rrs c1402t + tlyA WT	Composite	indeter	

Case	Medium	Less informative Mutation	ICV	More informative Mutation	ICV	MCV	BCV
2	H - composite	katG n.a. + inhA c-15t + mabA n.a. + furA n.a. + mshA n.a.	[green]	katG WT + inhA c-15t + mabA n.a. + furA n.a. + mshA n.a.	[red]	[red]	[yellow]
3	H - liquid	katG n.a. + inhA c-15t + mabA n.a. + furA n.a. + mshA n.a.	[green]	katG WT + inhA c-15t + mabA n.a. + furA n.a. + mshA n.a.	[red]	[red]	
4	H - solid	katG n.a. + inhA c-15t + mabA n.a. + furA n.a. + mshA n.a.	[green]	katG WT + inhA c-15t + mabA n.a. + furA n.a. + mshA n.a.	[yellow]	[yellow]	

Case	Drug	Gene	Mutation	Medium	ICV	BCV
5	R	*rpoB*	L533P	Composite	[red]	[yellow]
	R	*rpoB*	L533P	Liquid	indeter	
	R	*rpoB*	L533P	Solid	[yellow]	

Case	Drug	Gene	Mutation	DST	ICV	Interpretative ICV
6	R	*rpoB*	S531L	Liquid	[green]	[green]
	R	*rpoB*	L511P	Liquid	[black]	[black]
	R	*rpoB*	L511P+S531L	Liquid	n.a.	[green]

Case	Drug	Gene	Mutation	DST	MCV	Interpretative MCV
7	H	*katG+inhA*	katG S315N + inhA WT	Composite	[green]	[green]
	H	*katG+inhA*	katG WT + inhA c-15t	Composite	[red]	[red]
	H	*katG+inhA*	katG S315N + inhA c-15t	Composite	n.a.	[green]

Case	Drug	Gene	Mutation	Medium	ICV	Interpretative ICV	BCV	Interpretative BCV
			M515I	Composite	indeter	indeter		
			M515I	Solid	indeter	indeter	indeter	indeter
			M515I	Liquid	indeter	indeter		
			H526N	Composite	🔴	🔴		
**8**	R	*rpoB*	H526N	Solid	indeter	indeter	🔴	🔴
			H526N	Liquid	🔴	🔴		
			M515I+H526N	Composite	n.a.	n.a.		
			M515I+H526N	Solid	n.a.	n.a.	Indeter	🔴
			M515I+H526N	Liquid	n.a.	n.a.		

Case	Drug	Gene	Mutation	Medium	ICV	Interpretative ICV
			pooled frameshift and premature Stop	Composite	🟢	🟢
			V128ins	Composite	n.a.	🟢
			G24del	Composite	🟡	🟢
**9**	Z	*pncA*	A102T	Composite	n.a.	indeter
			A102T+G105ins	Composite	n.a.	🟢
			T168I	Composite	indeter	indeter
			W119Stop	Composite	n.a.	🟢
			W119Stop+T168I	Composite	n.a.	🟢

References

1. Hayden SR, Brown MD. Likelihood ratio: A powerful tool for incorporating the results of a diagnostic test into clinical decisionmaking. *Ann Emerg Med* 1999; **33**(5): 575-80.
2. Goodman SN. Toward evidence-based medical statistics. 2: The Bayes factor. *Ann Intern Med* 1999; **130**(12): 1005-13.
3. Brown MD, Reeves MJ. Evidence-based emergency medicine/skills for evidence-based emergency care. Interval likelihood ratios: another advantage for the evidence-based diagnostician. *Ann Emerg Med* 2003; **42**(2): 292-7.
4. Scorpio A, Zhang Y. Mutations in *pncA*, a gene encoding pyrazinamidase/nicotinamidase, cause resistance to the antituberculous drug pyrazinamide in tubercle bacillus. *Nat Med* 1996; **2**(6): 662-7.
5. DeBarber AE, Mdluli K, Bosman M, Bekker LG, Barry CE. Ethionamide activation and sensitivity in multidrug-resistant Mycobacterium tuberculosis. *Proc Natl Acad Sci U S A* 2000; **97**(17): 9677-82.
6. Okamoto S, Tamaru A, Nakajima C, et al. Loss of a conserved 7-methylguanosine modification in 16S rRNA confers low-level streptomycin resistance in bacteria. *Mol Microbiol* 2007; **63**(4): 1096-106.
7. Maus CE, Plikaytis BB, Shinnick TM. Mutation of tlyA confers capreomycin resistance in *Mycobacterium tuberculosis*. *Antimicrob Agents Chemother* 2005; **49**(2): 571-7.
8. Lew JM, Kapopoulou A, Jones LM, Cole ST. TubercuList--10 years after. *Tuberculosis (Edinb)* 2011; **91**(1): 1-7.
9. Telenti A, Imboden P, Marchesi F, et al. Detection of rifampicin-resistance mutations in *Mycobacterium tuberculosis*. *Lancet* 1993; **341**(8846): 647-50.
10. Campbell EA, Korzheva N, Mustaev A, et al. Structural mechanism for rifampicin inhibition of bacterial rna polymerase. *Cell* 2001; **104**(6): 901-12.
11. Maruri F, Sterling TR, Kaiga AW, et al. A systematic review of gyrase mutations associated with fluoroquinolone-resistant *Mycobacterium tuberculosis* and a proposed gyrase numbering system. *J Antimicrob Chemother* 2012; **67**(4): 819-31.
12. Köser CU, Niemann S, Summers DK, Archer JA. Overview of errors in the reference sequence and annotation of *Mycobacterium tuberculosis* H37Rv, and variation amongst its isolates. *Infect Genet Evol* 2012; **12**(4): 807-10.
13. Team RC. R: A language and environment for statistical computing. Vienna, Austria: R Foundation for Statistical Computing 2013.
14. Clopper CJ, Pearson ES. The use of confidence or fiducial limits illustrated in the case of the binomial. *Biometrika* 1934; **26**: 404-13.
15. Fisher RA. Confidence limits for a cross-product ratio. *Australian Journal of Statistics* 1962; **4**: 41.
16. Fay MP, Proschan MA, Brittain E. Combining one-sample confidence procedures for inference in the two-sample case. *Biometrics* 2015; **71**(1): 146-56.

Supplementary 6

RIFAMPICIN (R)

FIGURE S6.1: PRISMA DIAGRAM OF THE DATABASE QUERY

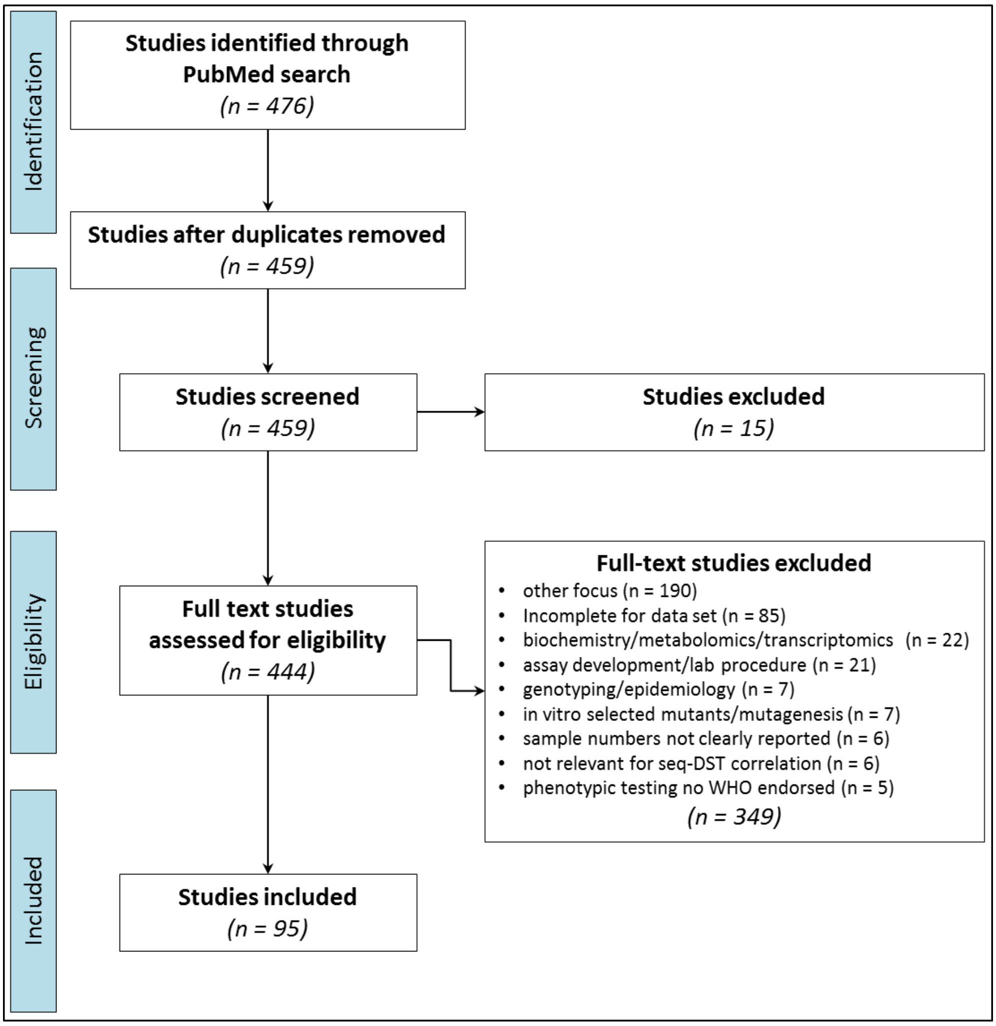

FIGURE S6.2: OVERVIEW OF THE CRITERIA FOR EXCLUDING PAPERS (N= 364)

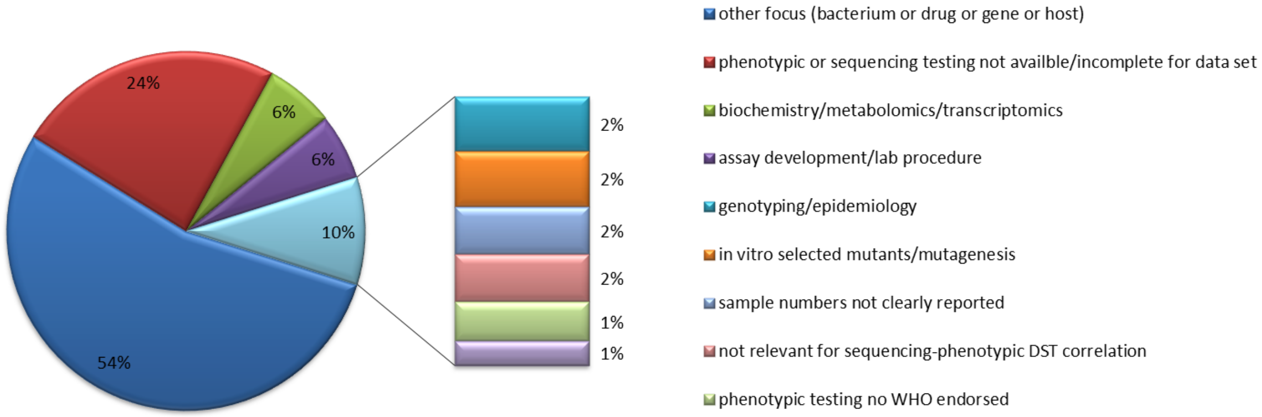

Of the 95 studies included, the type of laboratory performing the testing was only known for 36%. The breakdown concerning the knowledge of the laboratories in provided in Figure 3.

FIGURE 3: OVERVIEW OF THE STUDIES INCLUDED (N= 95)

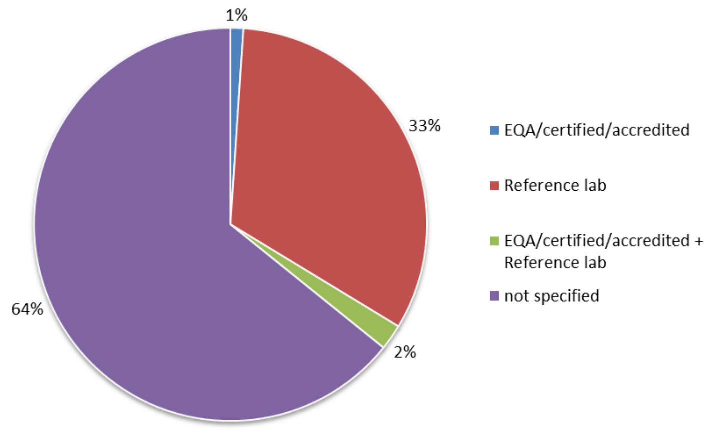

The number of countries of collection and isolation for isolates is reported in Figure 4. The geographical representativeness reported refer only to information provided in the studies and does not reflect speculation based upon the publication of the reference. However, given that many studies lack information on the origin of samples, it is conceivable that the geographic distribution of collection is broader than reported.

FIGURE S6.4: GLOBAL REPRESENTATIVENESS OF DATA SET

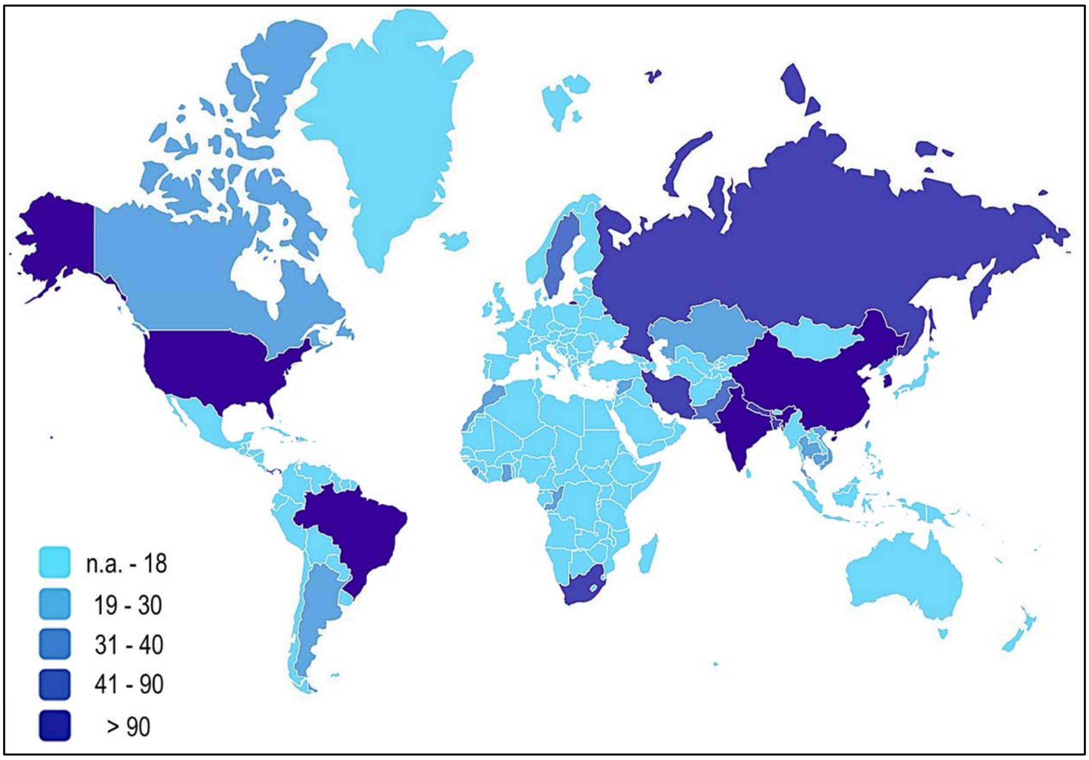

Geographical origin of clinical strains was available for 1851 isolates (22.9% of the total strains considered for analysis). An additional 264 clinical isolates were reported to be collected across different countries and/or regions, however specific details at the country level were not available.

TABLE S6.1: CLINICAL ISOLATES STRATIFIED BY PHENOTYPIC DST METHOD (CRITICAL CONCENTRATION DST) - RIFAMPICIN

**Testing method**	**Testing medium**		
	**Combined**	**Liquid**	**Solid**
BACTEC MGIT 960 - 1 µg/mL	2642	2642	0
BACTEC 460TB or MGIT 960 - 2 or 1 µg/mL	541	541	0
BACTEC 460TB - 2 µg/mL	189	189	0
Agar proportion - 1 µg/mL	881	0	881
LJ proportion - 40 µg/mL	7151	0	7151
Agar proportion - other	53	0	53
LJ absolute - 40 µg/mL	172	0	172
LJ proportion - other	98	0	98
not specified	1536	0	0
BACTEC MGIT 960 - 1 µg/mL or LJ proportion - 40 µg/mL	161	0	0
**Tot**	**13424**	**3372**	**8355**

ISONIAZID (H), ETHIONAMIDE (ETO), PROTHIONAMIDE (PTO)

FIGURE 5: PRISMA DIAGRAM OF THE DATABASE QUERY

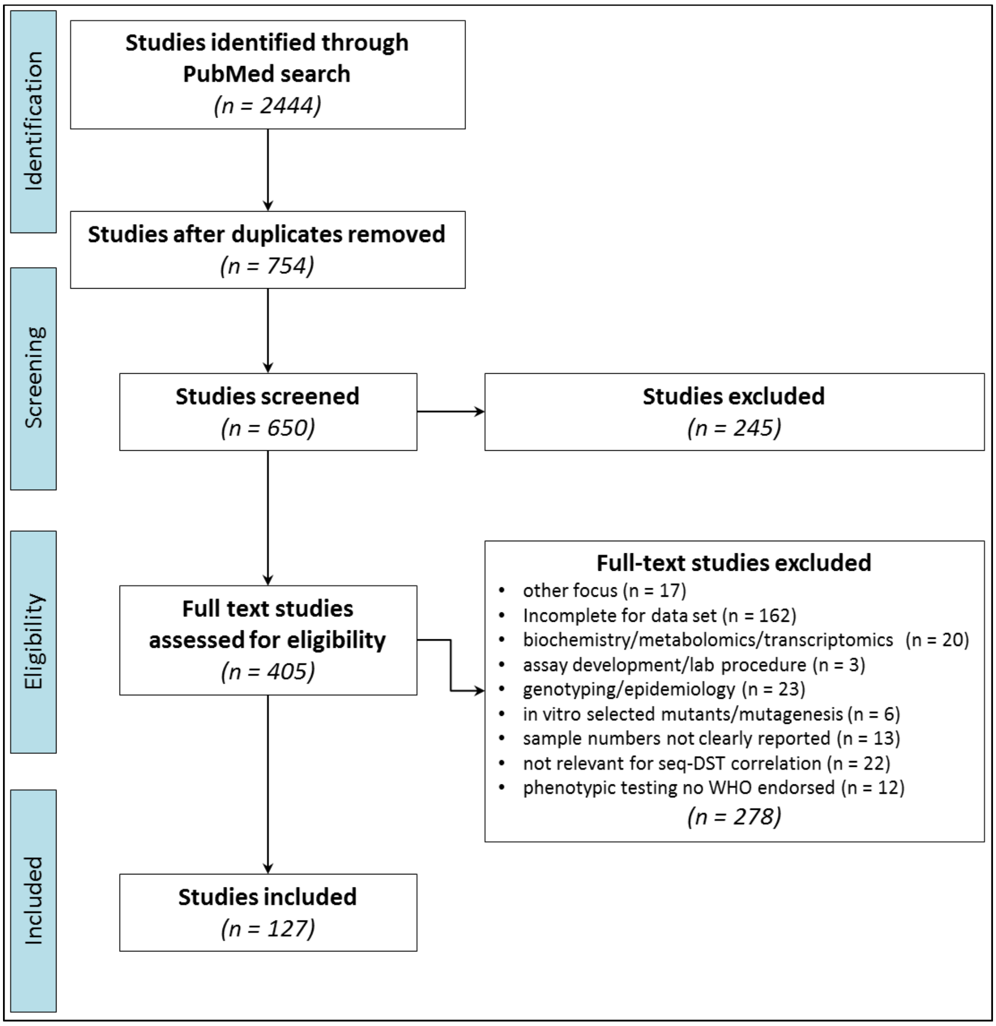

FIGURE S6.6: OVERVIEW OF THE CRITERIA FOR EXCLUDING PAPERS (N= 523)

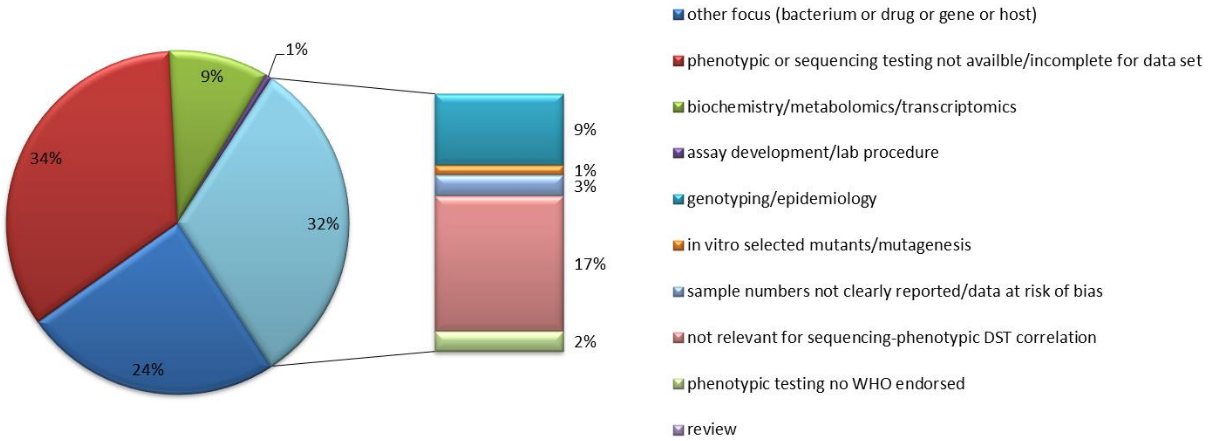

Of the 127 studies included, the quality of the laboratory performing the testing was known for 28% (Figure 7).

FIGURE S6.7: OVERVIEW OF THE STUDIES INCLUDED (N= 127)

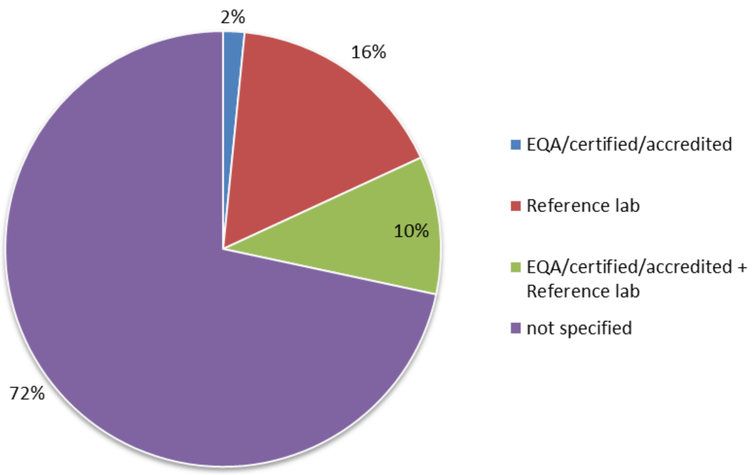

The number of countries of collection and isolation for isolates is reported in Figure 8. The geographical representativeness reported refer only to information provided in the studies and does not reflect speculation based upon the publication of the reference. However, given that many studies lack information on the origin of samples, it is conceivable that the geographic distribution of collection is broader than reported.

FIGURE 8: GLOBAL REPRESENTATIVENESS OF DATA SET

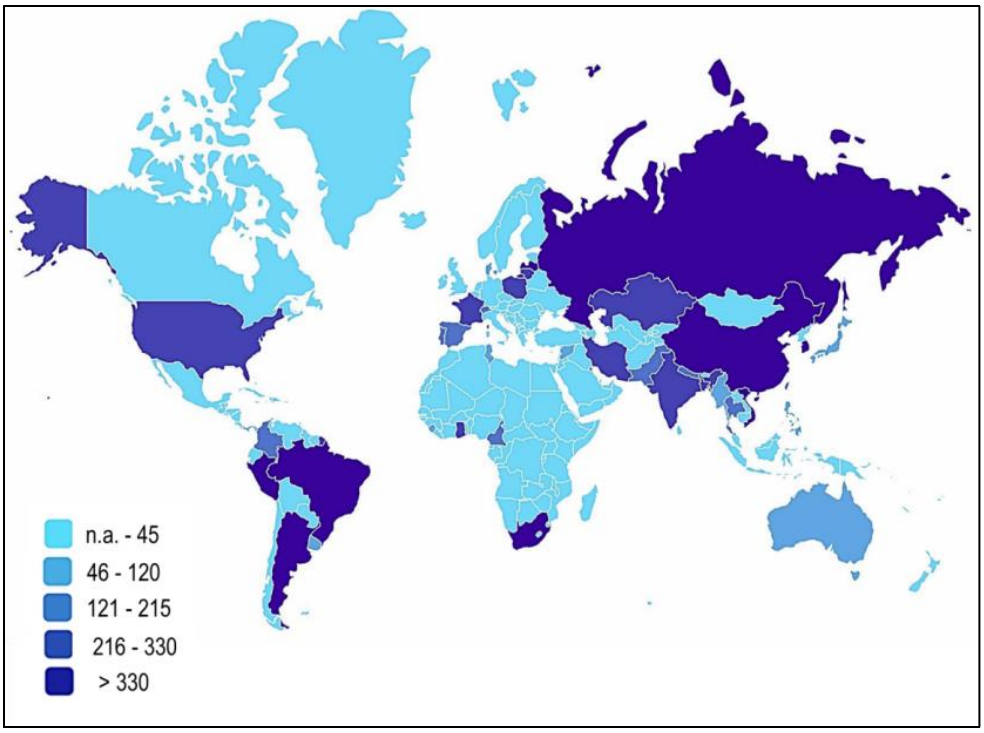

Geographical origin of clinical isolates was available for 11528 isolates (93% of the total isolates considered for analysis).

TABLE S6.2: CLINICAL ISOLATES STRATIFIED BY PHENOTYPIC DST METHOD (CRITICAL CONCENTRATION DST)

Medium	Drug	Method	*katG*		*inhA-mabA*						*furA*		*ethA*				*mshA*					
			H		H		ETO		PTO		H		ETO		PTO		H		ETO		PTO	
			DR	DS	DR	DS	DR	DS	DR	DS	DR	DS	DR	DS	DR	DS	DR	DS	DR	DS	DR	DS
Unknown	H	WHO recommended - not specified	536	27	461	21	-	-	-	-	-	-	-	-	-	-	-	-	-	-	-	-
Unknown	ETO	not specified 10 µg/mL	-	-	-	-	-	-	-	-	-	-	3	0	-	-	-	-	-	-	-	-
Unknown	ETO	not specified 2.5 µg/mL	-	-	-	-	-	-	-	-	-	-	8	3	-	-	-	-	-	-	-	-
Liquid	H	BACTEC MGIT960 - 0.1 µg/mL	1963	182	1595	233	-	-	-	-	1	0	-	-	-	-	10	64	-	-	-	-
Liquid	H	BACTEC MGIT960 - 0.1 µg/mL and 0.4 µg/mL	21	6	21	6	-	-	-	-	-	-	-	-	-	-	-	-	-	-	-	-
Liquid	H	BACTEC MGIT960 - not specified	97	0	96	0	-	-	-	-	-	-	-	-	-	-	-	-	-	-	-	-
Liquid	H	BACTEC460 - 0.1 µg/mL	907	49	694	27	-	-	-	-	38	0	-	-	-	-	-	-	-	-	-	-
Liquid	H	BACTEC460 - 0.2 µg/mL	34	11	34	11	-	-	-	-	-	-	-	-	-	-	-	-	-	-	-	-
Liquid	H	BACTEC460 - not specified	50	0	50	0	-	-	-	-	-	-	-	-	-	-	-	-	-	-	-	-
Liquid	H	BACTEC460 and BACTEC MGIT960 - both 0.1 µg/mL	82	43	82	43	-	-	-	-	-	-	-	-	-	-	-	-	-	-	-	-
Liquid	ETO	BACTEC MGIT960 - 5 µg/mL	-	-	-	-	-	-	-	-	-	-	23	33	-	-	-	-	-	-	-	-
Liquid	ETO	BACTEC MGIT960 - not specified	-	-	-	-	-	-	-	-	-	-	0	5	-	-	-	-	-	-	-	-
Liquid	ETO	BACTEC MGIT960 - 2.5 µg/mL	-	-	-	-	-	-	2	51	-	-	-	-	-	-	-	-	-	-	2	51
Liquid	ETO	BACTEC MGIT960 - not specified	-	-	-	-	-	-	5	13	-	-	-	-	5	13	-	-	-	-	-	-
Solid	H	7H10 agar proportion - 0.2 µg/mL	129	33	128	34	-	-	-	-	-	-	-	-	-	-	-	-	-	-	-	-
Solid	H	7H10 agar proportion - 0.2-1 µg/mL	1	0	1	0	-	-	-	-	1	0	-	-	-	-	-	-	-	-	-	-
Solid	H	7H10 agar proportion - 0.2-1-5 µg/mL	212	102	212	102	-	-	-	-	-	-	-	-	-	-	-	-	-	-	-	-
Solid	H	7H10 agar proportion - 1 µg/mL	11	0	-	-	-	-	-	-	-	-	-	-	-	-	-	-	-	-	-	-
Solid	H	absolute concentration - 0.2 µg/ml	52	28	40	6	-	-	-	-	-	-	-	-	-	-	-	-	-	-	-	-
Solid	H	agar proportion - 0.1-0.2-1.0-10.0 µg/ml	75	8	75	8	-	-	-	-	-	-	-	-	-	-	-	-	-	-	-	-
Solid	H	agar proportion - 0.2 µg/ml	429	0	422	0	-	-	-	-	-	-	-	-	-	-	-	-	-	-	-	-
Solid	H	agar proportion - 0.2-1.0 µg/ml	101	0	101	0	-	-	-	-	-	-	-	-	-	-	-	-	-	-	-	-
Solid	H	agar proportion - 0.2-1.0-5.0 µg/ml	446	0	446	0	-	-	-	-	-	-	-	-	-	-	-	-	-	-	-	-
Solid	H	agar proportion - 1 µg/mL	120	0	-	-	-	-	-	-	-	-	-	-	-	-	-	-	-	-	-	-
Solid	H	agar proportion - not specified	160	4	160	4	-	-	-	-	-	-	-	-	-	-	60	4	-	-	-	-
Solid	H	LJ absolute - not specified	47	59	47	59	-	-	-	-	-	-	-	-	-	-	-	-	-	-	-	-
Solid	H	LJ absolute concentration - 0.2-2 µg/mL	108	0	-	-	-	-	-	-	-	-	-	-	-	-	-	-	-	-	-	-
Solid	H	LJ absolute concentration - 1 µg/mL	429	0	412	0	-	-	-	-	-	-	-	-	-	-	-	-	-	-	-	-
Solid	H	LJ absolute concentration - 1-10 µg/ml	126	0	126	0	-	-	-	-	-	-	-	-	-	-	-	-	-	-	-	-
Solid	H	LJ proportion - 0.1.-0.2-1.0-10.0 µg/ml	95	0	33	0	-	-	-	-	-	-	-	-	-	-	-	-	-	-	-	-
Solid	H	LJ proportion - 0.2 µg/mL	3164	332	2404	279	-	-	-	-	-	-	-	-	-	-	100	50	-	-	-	-
Solid	H	LJ proportion - 0.2-1 µg/mL	141	311	141	311	-	-	-	-	110	211	-	-	-	-	-	-	-	-	-	-
Solid	H	LJ proportion - 0.2-2.0 µg/mL	51	0	-	-	-	-	-	-	-	-	-	-	-	-	-	-	-	-	-	-
Solid	H	LJ proportion - 0.25-1.0 µg/mL	32	65	-	-	-	-	-	-	-	-	-	-	-	-	-	-	-	-	-	-
Solid	H	LJ proportion - 1 µg/mL	344	38	234	20	-	-	-	-	-	-	-	-	-	-	-	-	-	-	-	-
Solid	H	LJ proportion - 2 µg/mL	42	0	-	-	-	-	-	-	-	-	-	-	-	-	-	-	-	-	-	-
Solid	H	LJ proportion - 5.0 µg/mL	70	0	70	0	-	-	-	-	-	-	-	-	-	-	-	-	-	-	-	-
Solid	H	LJ proportion - not specified	474	0	158	0	-	-	-	-	-	-	-	-	-	-	-	-	-	-	-	-
Solid	ETO	7H10 agar proportion - 5 µg/mL	-	-	-	-	22	40	-	-	-	-	-	-	-	-	-	-	-	-	-	-
Solid	ETO	7H10 proportion - 10 µg/mL	-	-	-	-	41	0	-	-	-	-	-	-	-	-	-	-	-	-	-	-
Solid	ETO	agar proportion - 20 µg/ml	-	-	-	-	75	8	-	-	-	-	-	-	-	-	-	-	-	-	-	-
Solid	ETO	agar proportion - 5 µg/ml	-	-	-	-	57	7	-	-	-	-	57	7	-	-	-	-	57	7	-	-
Solid	ETO	LJ proportion - 40 µg/mL	-	-	-	-	12	13	-	-	-	-	-	-	-	-	-	-	-	-	-	-
Solid	ETO	LJ proportion - 5 µg/mL	-	-	-	-	-	-	-	-	-	-	13	11	-	-	-	-	-	-	-	-

FLUOROQUINOLONES (OFX, LFX, MXF)

FIGURE S6.9: PRISMA DIAGRAM OF THE DATABASE QUERY

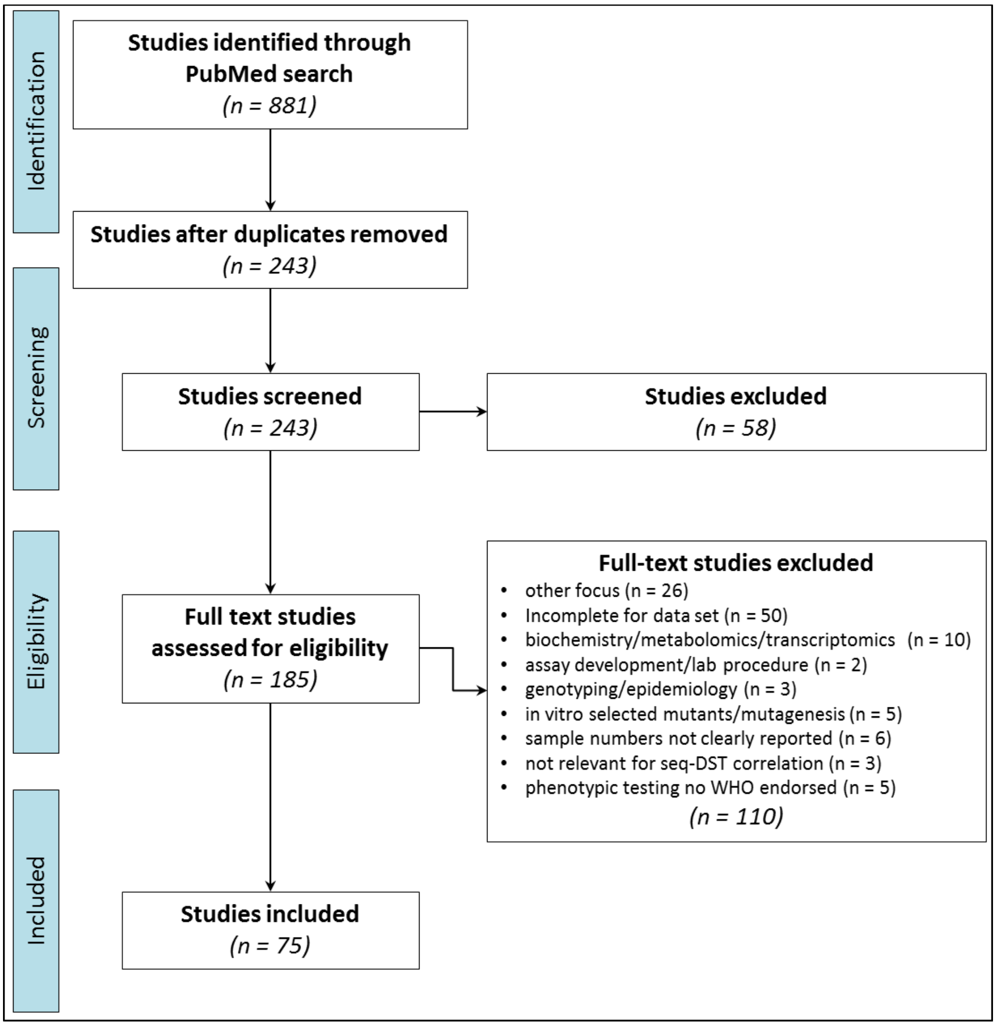

FIGURE S6.10: OVERVIEW OF THE CRITERIA FOR EXCLUDING PAPERS (N= 168)

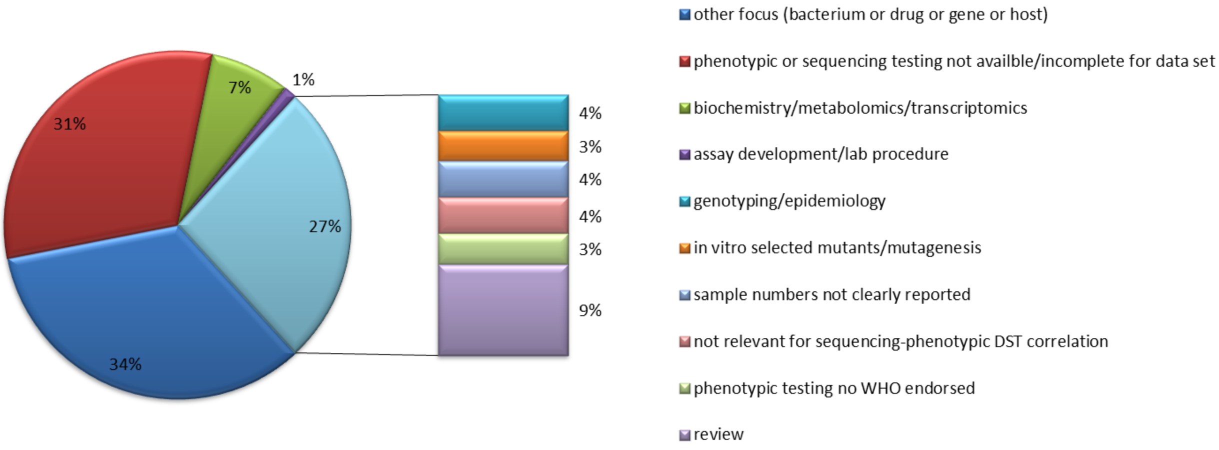

Of the 75 studies included, the type of laboratory performing the testing was only known for 33%. The breakdown concerning the knowledge of the laboratories in provided in Figure 11.

FIGURE S6.11: OVERVIEW OF THE STUDIES INCLUDED (N= 75)

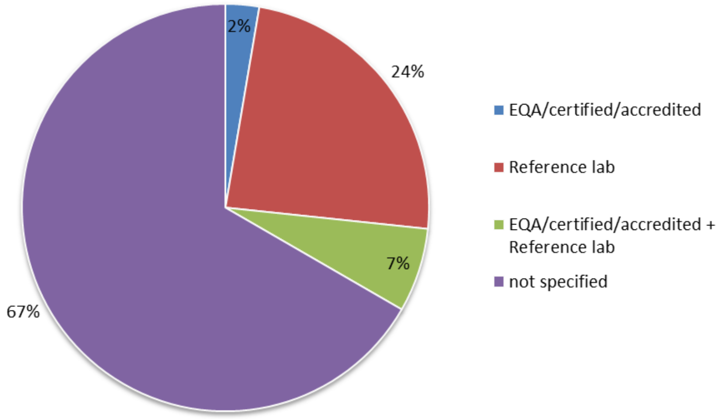

The number of countries of collection and isolation for isolates is reported in Figure 12. The geographical representativeness reported refer only to information provided in the studies and does not reflect speculation based upon the publication of the reference. However, given that many studies lack information on the origin of samples, it is conceivable that the geographic distribution of collection is broader than reported.

FIGURE S6.12: GLOBAL REPRESENTATIVENESS OF DATA SET

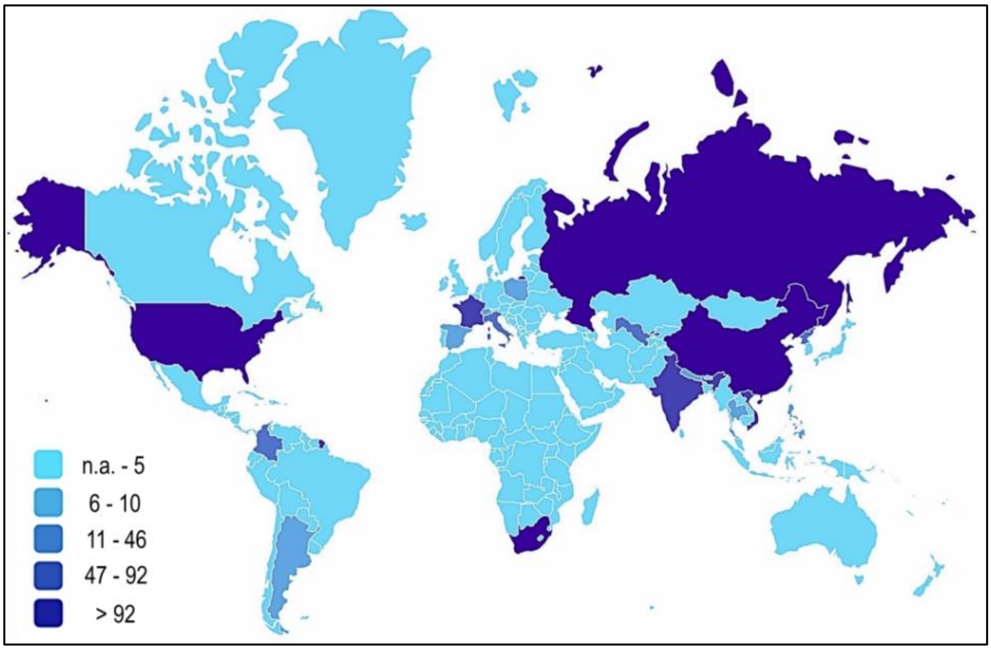

Geographical origin of clinical strains was available for 1171 isolates (13.4% of the total strains considered for analysis).

TABLE S6.3: CLINICAL ISOLATES STRATIFIED BY PHENOTYPIC DST METHOD (CRITICAL CONCENTRATION DST)

gyrA

Medium	Phenotypic testing method	MFX	OFX	LFX
unknown	BACTEC MGIT 960 or LJ proportion - 2 µg/mL	0	106	0
unknown	not specified	0	78	0
unknown	proportion and absolute - not specified	0	36	0
unknown	WHO recommended	0	247	0
liquid	BACTEC 460 - 2 µg/mL	0	26	0
liquid	BACTEC 460 - not specified	0	53	0
liquid	BACTEC MGIT 960 - 10 µg/mL	0	4	0
liquid	BACTEC MGIT 960 - 2 µg/mL	5	1566	4
liquid	BACTEC MGIT 960 - 2 and 10 µg/mL	0	21	0
liquid	BACTEC 460 - 0.5 µg/mL	22	0	0
liquid	BACTEC MGIT 960 - 0.25 µg/mL	371	0	0
liquid	BACTEC MGIT 960 - 1 µg/mL	150	0	0
liquid	BACTEC MGIT 960 - 0.125 µg/mL	14	0	0
liquid	BACTEC MGIT 960 - 0.5 µg/mL	14	0	0
liquid	BACTEC MGIT 960 - 1.5 µg/mL	0	0	12
solid	7H10 submerged-disk proportion method - 2 µg/mL	0	102	0
solid	7H11 agar proportion - 2 µg/mL	0	306	0
solid	7H11 multiple proportion - 2 µg/mL	0	10	10
solid	LJ absolute - 10 µg/mL	0	133	0
solid	LJ absolute - 2 µg/mL	0	769	0
solid	LJ proportion - 2 µg/mL	0	2021	226
solid	7H10 agar proportion - 0.5 µg/mL	152	0	0
solid	7H10 agar proportion - 1 µg/mL	170	0	42
solid	7H11 agar proportion - 0.5 µg/mL	40	0	0
solid	7H11 multiple proportion - 0.5 µg/mL	10	0	0
solid	LJ proportion - 0.75 µg/mL	68	0	0
solid	7H10 agar proportion - 2 µg/mL	3	433	0
solid	LJ proportion - 1 µg/mL	0	0	93
solid	LJ proportion - 10 µg/mL	0	0	62
	**Tot**	**1019**	**5911**	**449**

gyrB

Medium	Phenotypic testing method	MFX	OFX	LFX
unknown	not specified	0	78	0
unknown	proportion and absolute - not specified	0	36	0
liquid	BACTEC MGIT 960 - 10 µg/mL	0	4	0
liquid	BACTEC MGIT 960 - 2 µg/mL	0	861	0
liquid	BACTEC MGIT 960 - 2 and 10 µg/mL	0	21	0
liquid	BACTEC MGIT 960 - 0.25 µg/mL	120	0	0
liquid	BACTEC MGIT 960 - 1 µg/mL	150	0	0
liquid	BACTEC MGIT 960 - 0.125 µg/mL	14	0	0
liquid	BACTEC MGIT 960 - 0.5 µg/mL	14	0	0
solid	7H11 agar proportion - 2 µg/mL	0	72	0
solid	7H11 multiple proportion - 2 µg/mL	0	10	10
solid	LJ absolute - 10 µg/mL	0	26	0
solid	LJ absolute - 2 µg/mL	0	111	0
solid	LJ proportion - 2 µg/mL	0	1442	11
solid	7H10 agar proportion - 0.5 µg/mL	149	0	0
solid	7H10 agar proportion - 1 µg/mL	170	0	42
solid	7H11 agar proportion - 0.5 µg/mL	40	0	0
solid	7H11 multiple proportion - 0.5 µg/mL	10	0	0
solid	LJ proportion - 0.75 µg/mL	68	0	0
solid	7H10 agar proportion - 2 µg/mL	0	417	0
solid	LJ proportion - 1 µg/mL	0	0	93
solid	LJ proportion - 10 µg/mL	0	0	62
	**Tot**	**735**	**3078**	**218**

PYRAZINAMIDE (Z)

FIGURE S6.13: PRISMA DIAGRAM OF THE DATABASE QUERY

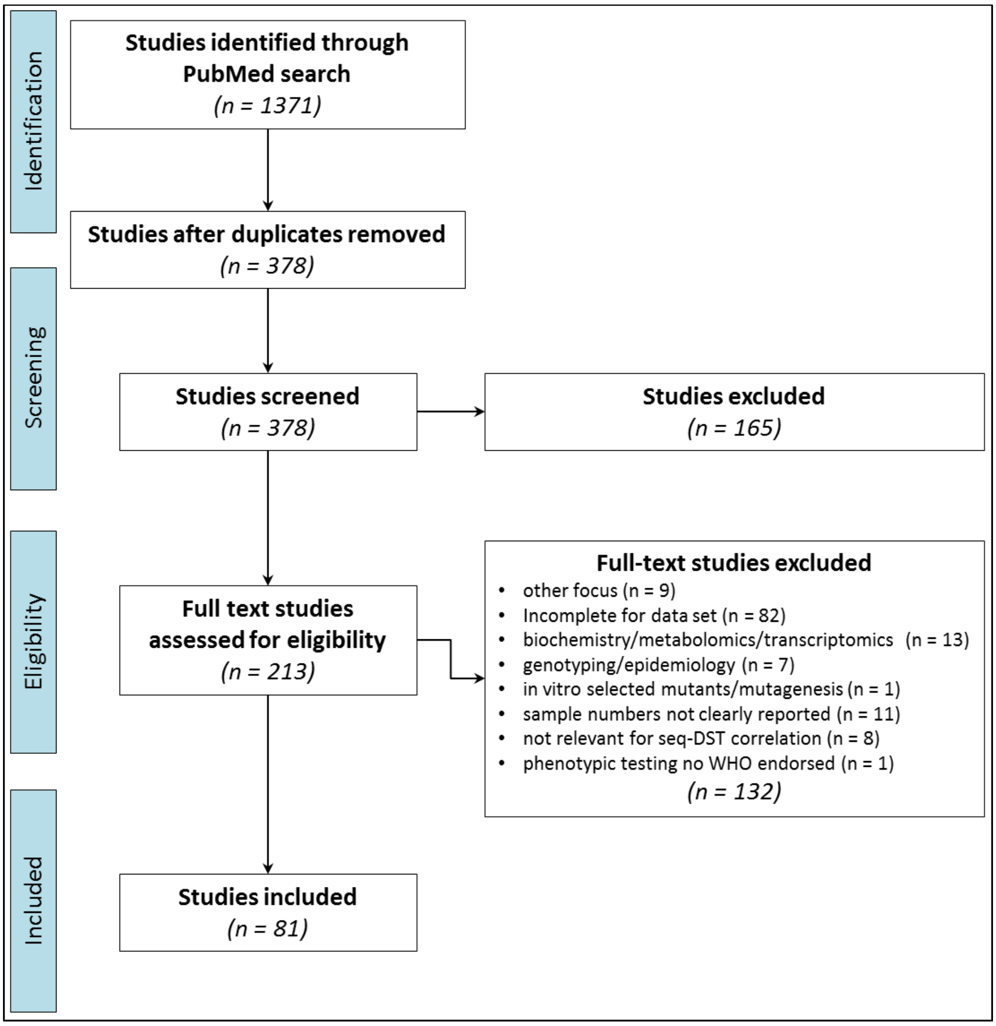

FIGURE S6.14: OVERVIEW OF THE CRITERIA FOR EXCLUDING PAPERS (N= 297)

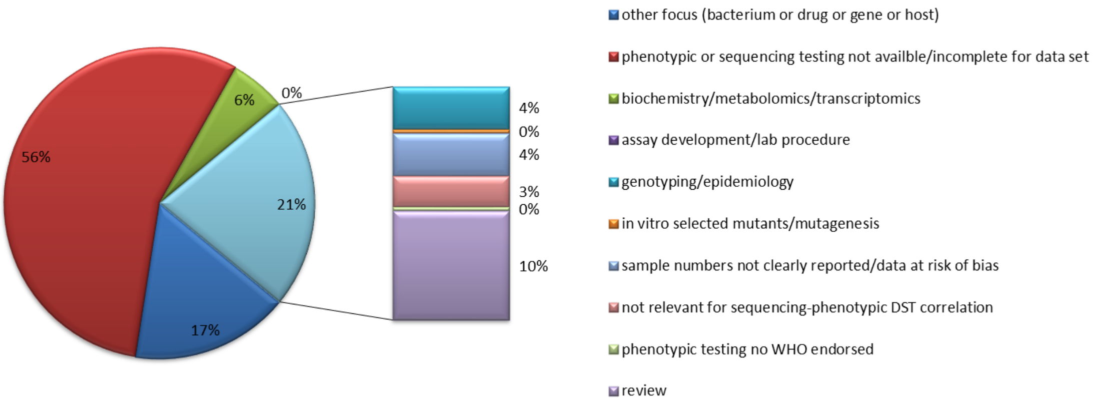

Of the 81 studies included, the type of laboratory performing the testing was only known for 33%. The breakdown concerning the knowledge of the laboratories in provided in Figure 15.

FIGURE S6.15: OVERVIEW OF THE STUDIES INCLUDED (N= 81)

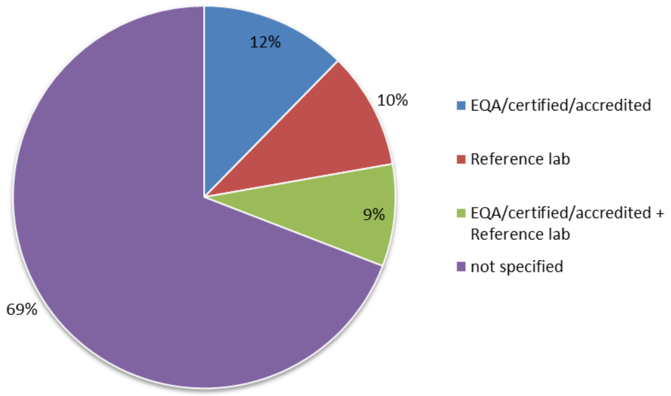

The number of countries of collection and isolation for isolates is reported in Figure 16. The geographical representativeness reported refer only to information provided in the studies and does not reflect speculation based upon the publication of the reference. However, given that many studies lack information on the origin of samples, it is conceivable that the geographic distribution of collection is broader than reported.

FIGURE S6.16: GLOBAL REPRESENTATIVENESS OF DATA SET

(Phenotypic DST on liquid media) (Wayne pyrazinamidase activity assay)

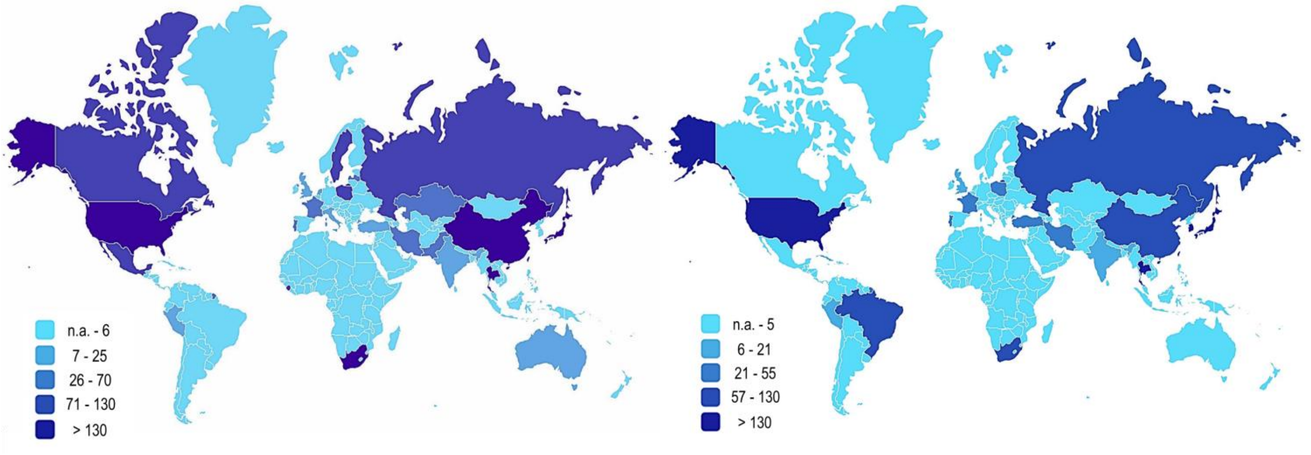

Geographical origin of clinical strains was available for 2252 isolates for liquid phenotypic DST (46% of the total strains considered for the analysis) and 1447 isolates for Wayne assay (83% of the total strains considered for analysis).

TABLE S6.4: CLINICAL ISOLATES STRATIFIED BY PHENOTYPIC DST METHOD (CRITICAL CONCENTRATION DST) - PYRAZINAMIDE

**Phenotypic testing method**	**DR**	**DS**
7H12B broth - 100 µg/mL	6	5
7H9 broth - 100 µg/mL	82	18
BACTEC 460 - 100 µg/mL	534	583
BACTEC MGIT960 - 100 µg/mL	1796	1925
**tot**	**2418**	**2531**

AMINOGLYCOSIDES (STREPTOMYCIN (S), CAPREOMYCIN (CM), AMIKACIN (AM), KANAMYCIN (KM))

FIGURE S6.17: RESULTS FROM DATABASE QUERY

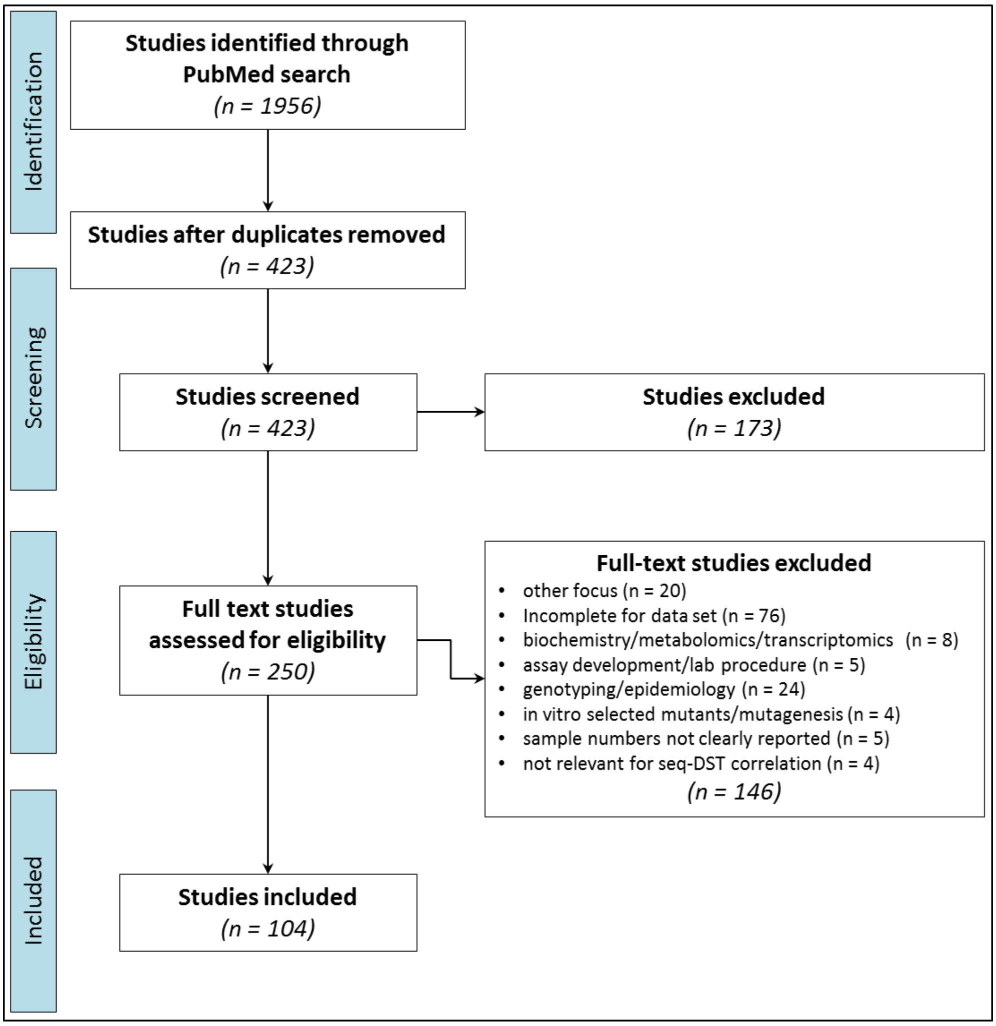

FIGURE S6.18: OVERVIEW OF THE STUDIES INCLUDED (N= 104)

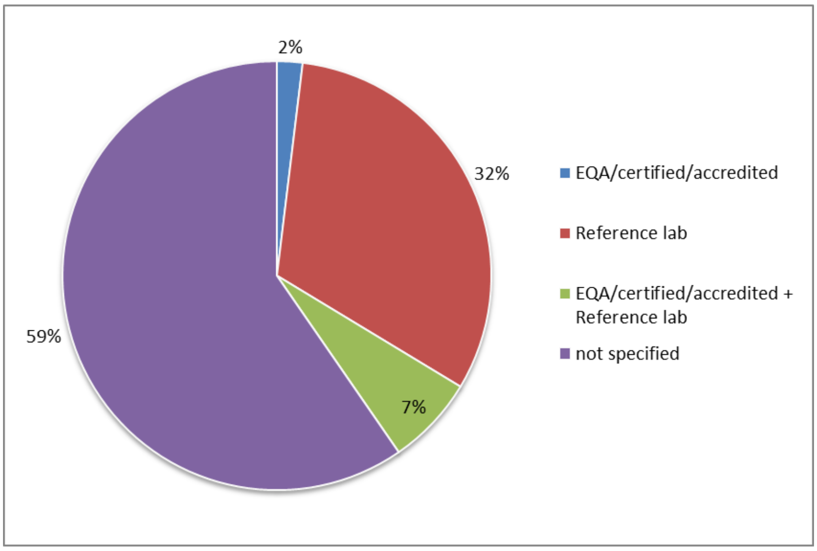

The number of countries of collection and isolation for isolates is reported in Figure 19. The geographical representativeness reported refer only to information provided in the studies and does not reflect speculation based upon

the publication of the reference. However, given that many studies lack information on the origin of samples, it is conceivable that the geographic distribution of collection is broader than reported.

FIGURE S6.19: GLOBAL REPRESENTATIVENESS OF DATA SET

AM

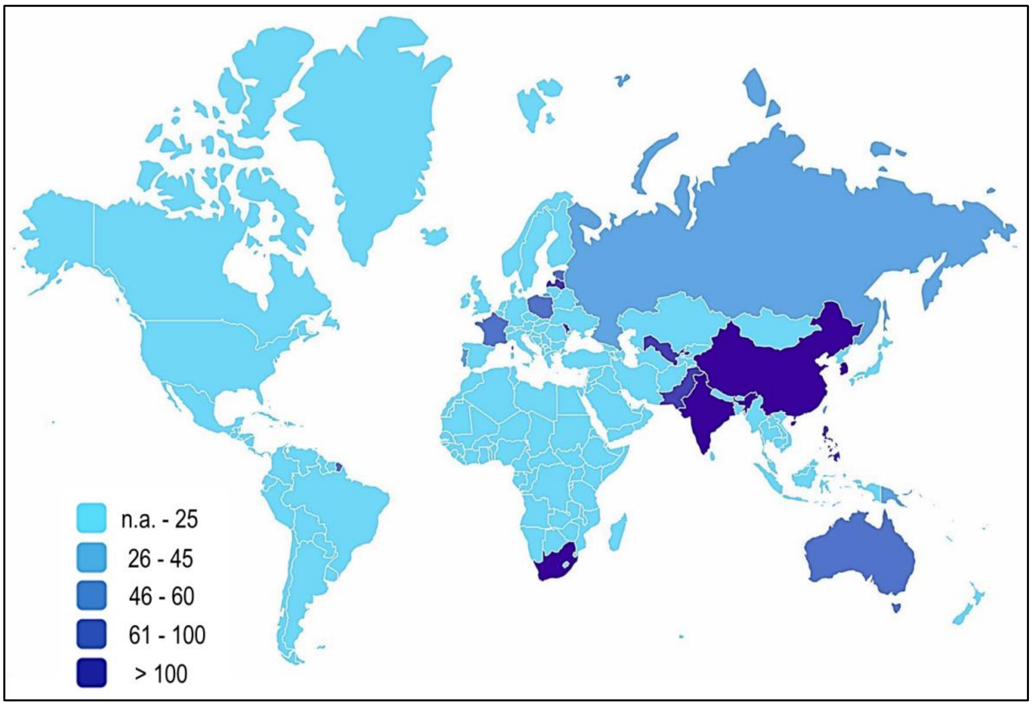

CM

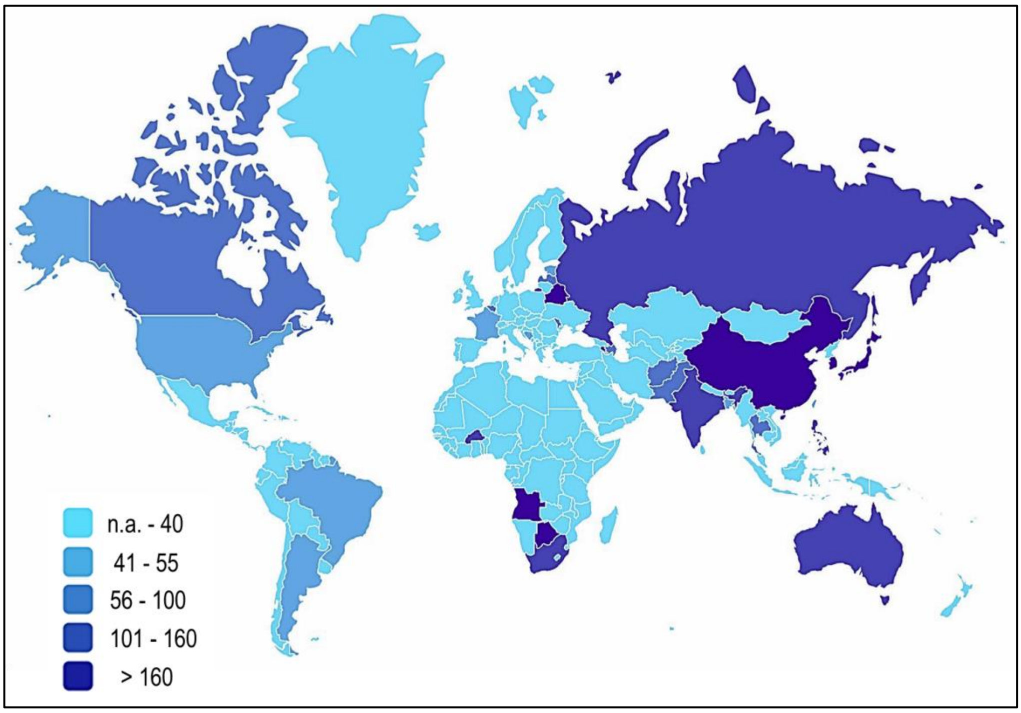

KM

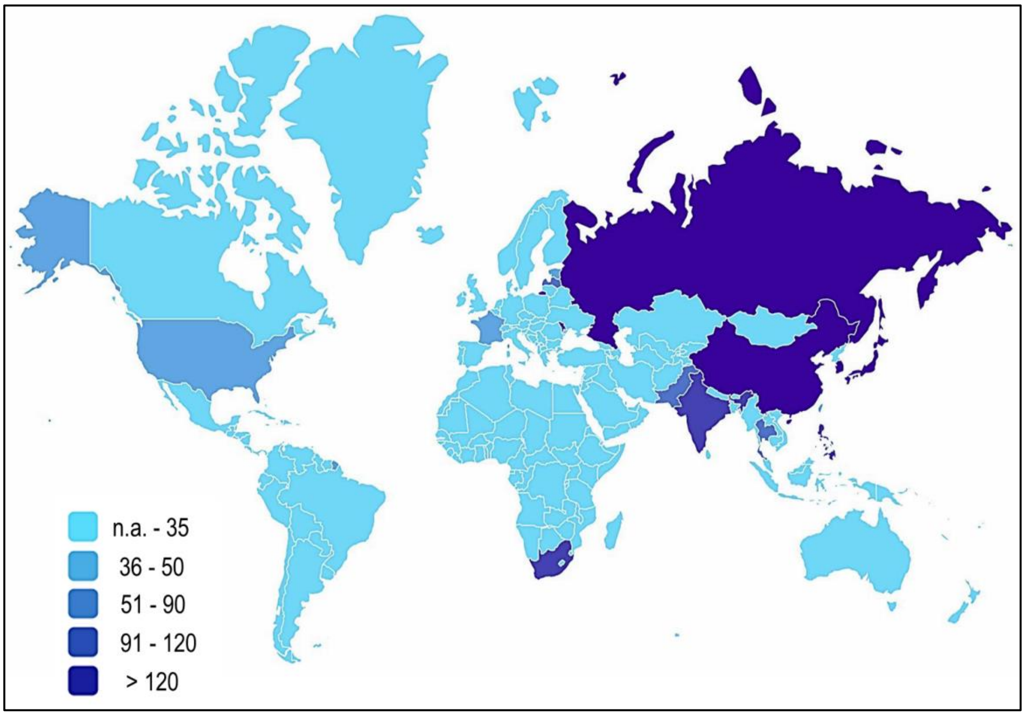

S

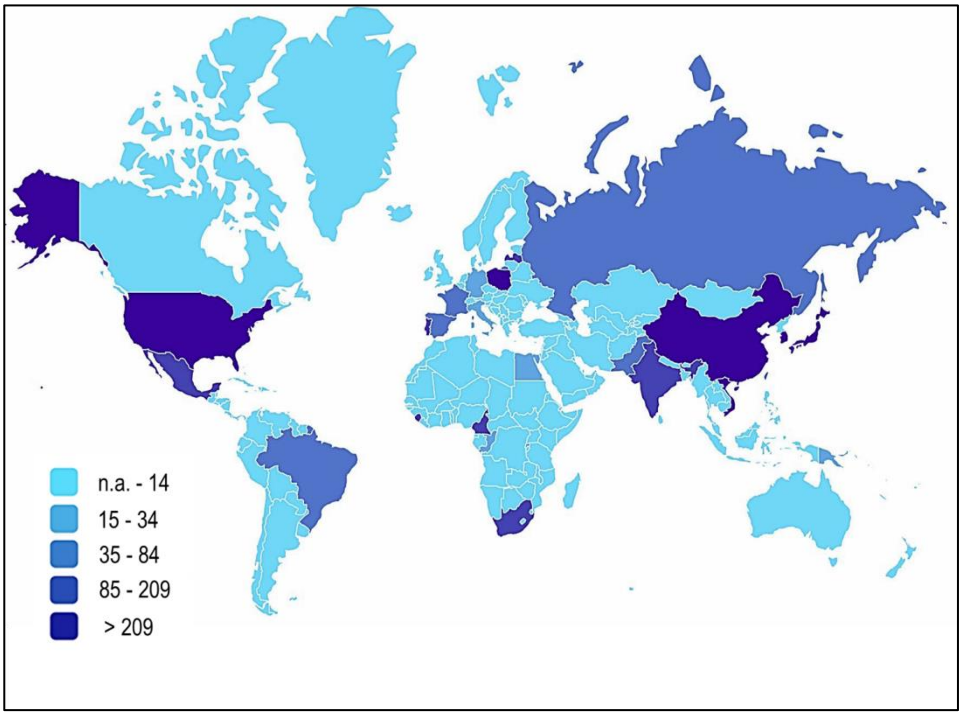

TABLE S6.5: CLINICAL ISOLATES STRATIFIED BY PHENOTYPIC DST METHOD (CRITICAL CONCENTRATION DST)

Method	Medium	Drug	*rrs*		*tlyA*		*eis*		*rpsL*		*gidB*		*tap*		*whib7*	
			DR	DS	DR	DS	DR	DS	DR	DS	DR	DS	DR	DS	DR	DS
unknown	Unknown	AM	28	29	0	0	0	0	0	0	0	0	0	0	0	0
WHO endorsed	Unknown	AM	44	43	44	43	0	0	0	0	0	0	0	0	0	0
BACTEC 460	Liquid	AM	1	7	0	0	0	0	1	7	0	0	0	0	0	0
BACTEC 460 - 1 µg/mL	Liquid	AM	17	58	14	12	14	12	0	0	0	0	0	0	0	0
BACTEC MGIT 960	Liquid	AM	11	8	0	0	0	0	11	8	0	0	0	0	0	0
BACTEC MGIT 960 - 1 µg/mL	Liquid	AM	95	239	0	0	0	0	0	0	0	0	0	0	0	0
BACTEC MGIT 960 - 1.0 µg/mL	Liquid	AM	4	34	4	7	4	7	0	27	0	27	0	0	0	0
BACTEC MGIT 960 - 1.5 µg/mL	Liquid	AM	17	74	0	0	17	74	0	0	0	0	0	0	0	0
microplate - 1 µg/mL	Liquid	AM	1	12	0	0	1	12	0	0	0	0	0	0	0	0
7H10 agar proportion - 4 µg/mL	Solid	AM	132	257	62	13	62	12	0	0	0	0	0	0	0	0
7H10 agar proportion - 4 µg/mL and 8 µg/mL	Solid	AM	84	155	0	0	0	0	0	0	0	0	0	0	0	0
7H10 agar proportion - 5 µg/mL	Solid	AM	37	0	37	0	0	0	37	0	37	0	0	0	0	0
7H10 agar proportion - 6 µg/mL	Solid	AM	28	10	0	0	0	0	0	0	0	0	0	0	0	0
7H11 agar proportion - 4 µg/mL	Solid	AM	30	20	30	20	0	0	0	0	0	0	0	0	0	0
7H11 agar proportion - 6 µg/mL	Solid	AM	50	0	0	0	0	0	0	0	0	0	0	0	0	0
agar proportion - 20 µg/mL	Solid	AM	20	27	0	0	20	27	0	0	0	0	0	0	0	0
LJ method; 40 µg/mL	Solid	AM	113	87	103	54	113	87	113	87	113	87	0	0	0	0
LJ proportion - 30µg/mL	Solid	AM	35	173	35	173	0	0	0	0	0	0	0	0	0	0
LJ proportion - 40 µg/mL	Solid	AM	46	60	0	0	0	0	0	0	0	0	0	0	0	0
LJ proportion method; 40 µg/mL	Solid	AM	16	3	0	0	0	0	0	0	0	0	0	0	0	0

Method	Medium	Drug	*rrs*		*tlyA*		*eis*		*rpsL*		*gidB*		*tap*		*whib7*	
			DR	DS	DR	DS	DR	DS	DR	DS	DR	DS	DR	DS	DR	DS
unknown	Unknown	CM	17	40	0	0	0	0	0	0	0	0	0	0	0	0
WHO endorsed	Unknown	CM	43	44	43	44	0	0	0	0	0	0	0	0	0	0
BACTEC 460 - 1.25 µg/mL	Liquid	CM	26	0	26	0	26	0	0	0	0	0	0	0	0	0
BACTEC 460 or BACTEC MGIT 960 - 460: 1.25 µg/mL, 960: 3 µg/mL	Liquid	CM	10	91	0	0	10	91	0	0	0	0	0	0	0	0
BACTEC MGIT 960	Liquid	CM	11	8	0	0	0	0	11	8	0	0	0	0	0	0
BACTEC MGIT 960 - 1 .25 µg/mL	Liquid	CM	4	25	4	25	4	25	4	25	0	0	0	0	0	0
BACTEC MGIT 960 - 2.5 µg/mL	Liquid	CM	57	94	7	4	7	4	0	27	0	27	0	0	0	0
REMA - 2.5 µg/mL	Liquid	CM	0	21	0	0	0	0	0	21	0	0	0	0	0	0
7H10 agar proportion - 4 µg/mL	Solid	CM	66	86	66	86	66	86	0	0	0	0	0	0	0	0
7H11 agar proportion - 30 µg/mL	Solid	CM	82	678	82	678	0	0	0	0	0	0	0	0	0	0
agar proportion - 40 µg/mL	Solid	CM	24	23	0	0	24	23	0	0	0	0	0	0	0	0
LJ absolute - 40 µg/mL	Solid	CM	13	0	0	0	0	0	0	0	0	0	0	0	0	0
LJ proportion - 40 µg/mL	Solid	CM	187	349	129	282	100	57	100	57	100	57	0	0	0	0
LJ proportion method - 20 µg/mL	Solid	CM	11	41	0	0	0	0	11	41	0	0	0	0	0	0
7H10 agar proportion - 6 µg/mL	Solid	CM	22	16	0	0	0	0	0	0	0	0	0	0	0	0
7H10 agar proportion - 10 µg/mL	Solid	CM	155	289	119	259	56	18	14	23	14	23	0	0	0	0

Method	Medium	Drug	*rrs*		*tlyA*		*eis*		*rpsL*		*gidB*		*tap*		*whib7*	
			DR	DS	DR	DS	DR	DS	DR	DS	DR	DS	DR	DS	DR	DS
BACTEC 460 - 5 µg/mL	Liquid	KM	27	12	24	2	24	2	3	10	0	0	0	0	0	0
BACTEC 460 or BACTEC MGIT 960 - 460: 5 µg/mL, 960: 2.5 µg/mL	Liquid	KM	30	71	0	0	30	71	0	0	0	0	0	0	0	0
BACTEC MGIT 960	Liquid	KM	22	7	22	7	22	7	22	7	0	0	0	0	0	0
BACTEC MGIT 960 - 2.5 µg/mL	Liquid	KM	2	4	2	4	2	4	0	0	0	0	0	0	0	0
BACTEC MGIT 960 - 5 µg/mL	Liquid	KM	0	27	0	0	0	0	0	27	0	27	0	0	0	0
REMA - 2.5 µg/mL	Liquid	KM	3	18	0	0	0	0	3	18	0	0	0	0	0	0
7H10 agar proportion - 4 µg/mL	Solid	KM	216	250	105	47	152	248	0	0	0	0	0	0	0	0
7H10 agar proportion - 5 µg/mL	Solid	KM	63	12	63	12	63	11	0	0	0	0	0	0	0	0
7H10 agar proportion - 6 µg/mL	Solid	KM	104	27	66	27	67	27	37	0	37	0	0	0	29	27
7H10 agar proportion - not specified	Solid	KM	14	29	0	0	0	0	14	29	0	0	0	0	0	0
7H11 agar proportion - 30 µg/mL	Solid	KM	0	0	0	0	114	646	0	0	0	0	0	0	0	0
7H11 agar proportion - 6 µg/mL	Solid	KM	50	0	0	0	0	0	0	0	0	0	0	0	0	0
LJ absolute - 30 µg/mL	Solid	KM	13	0	0	0	0	0	0	0	0	0	0	0	0	0
LJ absolute - 40 µg/mL	Solid	KM	20	86	20	86	0	0	20	86	20	85	0	0	0	0
LJ method - 20 µg/mL	Solid	KM	10	0	0	0	0	0	10	0	0	0	0	0	0	0
LJ proportion - 30 µg/mL	Solid	KM	135	266	0	0	117	265	33	25	33	25	0	0	0	0
LJ proportion - 40 µg/mL	Solid	KM	110	47	110	47	110	47	110	47	110	47	0	0	0	0
LJ proportion method - 20 µg/mL	Solid	KM	10	42	0	0	0	0	10	42	0	0	0	0	0	0

Method	Medium	Drug	*rrs*		*tlyA*		*eis*		*rpsL*		*gidB*		*tap*		*whib7*	
			DR	DS	DR	DS	DR	DS	DR	DS	DR	DS	DR	DS	DR	DS
BACTEC 460 or 7H10 agar proportion - BACTEC: 6 µg/mL, 7H10: 2 and 10 µg/mL	Unknown	S	65	50	0	0	0	0	70	59	0	0	0	0	0	0
not specified	Unknown	S	115	100	0	0	0	0	115	100	0	0	0	0	0	0
proportion - 2 µg/mL	Unknown	S	31	0	0	0	0	0	31	0	0	0	0	0	0	0
proportion method	Unknown	S	0	0	0	0	0	0	7	10	0	0	0	0	0	0
unknown	Unknown	S	0	0	0	0	0	0	104	20	0	0	0	0	0	0
BACTEC 460	Liquid	S	54	16	0	0	0	0	44	16	0	0	0	0	0	0
BACTEC 460 or BACTEC MGIT 960 - 460: 2-6 mg/ml; MGIT:1-4 mg/ml	Liquid	S	69	0	0	0	0	0	69	0	0	0	0	0	0	0
BACTEC 12B	Liquid	S	0	0	0	0	0	0	61	3	0	0	0	0	0	0
BACTEC 460 - 10 µg/mL	Liquid	S	4	9	0	0	0	0	4	9	0	0	0	0	0	0
BACTEC 460 - 2 µg/mL	Liquid	S	148	24	26	7	26	7	193	113	0	0	0	0	0	0
BACTEC 460 - 6 µg/mL	Liquid	S	5	16	0	0	0	0	5	16	0	0	0	0	0	0
BACTEC 460 - not specified	Liquid	S	102	46	0	0	0	0	104	46	0	0	0	0	0	0
BACTEC MGIT 960	Liquid	S	13	6	0	0	0	0	13	6	0	0	0	0	0	0
BACTEC MGIT 960 - 1 µg/mL	Liquid	S	76	21	10	1	10	1	212	92	9	18	0	0	0	0
BACTEC MGIT 960 - 1 µg/mL; 4 µg/mL	Liquid	S	31	11	31	11	31	11	31	11	0	0	0	0	0	0
BACTEC MGIT 960 - not specified	Liquid	S	121	108	0	0	0	0	121	108	0	0	0	0	0	0
REMA - 1 µg/mL	Liquid	S	49	81	0	0	0	0	49	81	40	66	0	0	0	0
7H10 agar proportion - 2 µg/mL	Solid	S	69	10	50	6	0	0	61	12	32	5	0	0	0	0
7H10 agar proportion - not specified	Solid	S	73	108	0	0	0	0	73	108	0	0	0	0	0	0
LJ absolute - 4 µg/mL	Solid	S	38	1	0	0	0	0	66	0	0	0	0	0	0	0
LJ method - 4 µg/mL	Solid	S	37	19	0	0	37	19	37	19	37	19	0	0	0	0
LJ proportion - 4 µg/mL	Solid	S	359	237	0	0	0	0	465	213	194	168	0	0	0	0
LJ proportion - 4 µg/mL and 8 µg/mL	Solid	S	40	57	0	0	0	0	40	57	40	57	0	0	0	0
LJ proportion - not specified	Solid	S	0	0	0	0	0	0	7	3	0	0	0	0	0	0
LJ proportion -4µg/mL	Solid	S	27	100	0	0	0	0	27	100	27	100	0	0	0	0
LJ proportion method - 4 µg/mL	Solid	S	38	14	0	0	0	0	38	14	0	0	0	0	0	0

Figure S6.20. Distribution of genetic variants in resistant (panel A, left) and susceptible (panel B, right) clinical isolates across the different drugs considered.

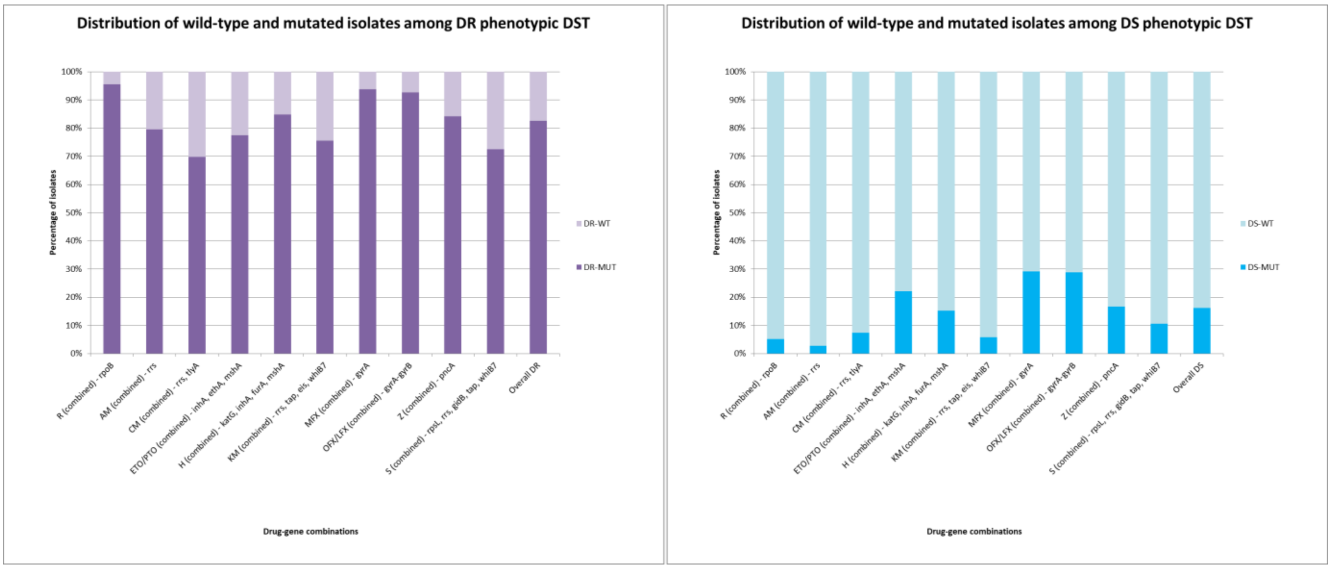

Supplementary 11

Table S11.1. Comparison of the list of mutations predictive of resistance described in this study and in Farhat *et al*[1]. Genetic variants common to both studies are highlighted in yellow. Association between mutation and drug resistance confirmed by False Discovery Rate corrected p-values and used for comparing performance characteristics are reported in bold. R: rifampicin; AM: amikacin; CM: capreomycin; ETO/PTO: ethionamide and/or prothionamide; H: isoniazid; KM: kanamycin; MFX: moxifloxacin; OFX/LFX: ofloxacin and/or levofloxacin; Z: pyrazinamide; S: streptomycin.

**Drug (phenotypic testing)**		**Gene**	**This study<br>(high, moderate, and minimal confidence mutations)**	**Farhat *et al* 2016<br>(Minimum list of predictive variables)**
First-line	R	*rpoB*	F505V+D516Y, **L511P**, S512T, Q513H+L533P, Q513-F514ins, **Q513K**, **Q513L**, **Q513P**, **F514dupl,** M515I+D516Y, **D516A**, **D516F**, **D516G**, D516G+L533P, D516ins, D516N, **D516V** , **D516Y**, Del N518, **S522L**, **S522Q**, **H526C**, **H526D**, H526F, **H526G**, **H526L**, **H526N**, H526P, **H526R**, **H526Y** **S531F**, **S531L**, S531Q, **S531W**, S531Y, **L533P**, **I572F**, D626E	S531L, D516V, H526Y, H526L, H526D, D516Y, L533P, Q513H, L511P, S531Stop, I572F, V146F
	H	*inhA-mabA*<br>*katG*<br>*embB*<br>*iniB*<br>*kasA*<br>*ahpC*	**c-15t**<br>S315I, **S315N**, **S315T**, Frameshifts and premature Stop codons<br>(Genomic locus not considered)<br>(Genomic locus not considered)<br>(Genomic locus not considered)<br>(Genomic locus not considered)	g-17t, c-15t, t-8c, t-8g, S94A,<br>S315T, S315N, S315R<br>M306I, M306V, G406A, G406S, S297A, c-12t<br>A70T<br>G269S<br>V49G
Second-line (group A)	MFX	*gyrA*	G88C, **A90V**, **S91P**, **D94A**, **D94G**, **D94N**, **D94Y**	(Drug not considered)
	OFX/LFX	*gyrA*<br>*gyrB*	**G88A**, **G88C,** D89N, **A90V**, **S91P**, **D94A**, **D94G**, **D94H**, **D94N**, **D94Y**<br>**A504V**, E459K	G88C, D89N, A90V, S91P, D94Y, D94A, D94G, D94H, D94N<br>N538T
Second-line (group B)	AM	*rrs*	**a1401g**, **g1484t**	a1401g, a514c
	KM	*eis*<br>*rrs*<br>*tlyA*	**c-14t**, **g-10a**, c-12t, g-37t<br>**a1401g**, c1402t, g1484t<br>(Genomic locus not considered)	(Genomic locus not considered)<br>a1401g<br>Stop269W
	CM	*rrs*<br>*tlyA*	**a1401g**, **c1402t**, **g1484t**<br>**N236K, Frameshifts and premature Stop codons**	a1401g, t1264g, a908c, g1315a, g1498t<br>-
	S	*rpsL*<br>*rrs*<br>*gidB*	**K43R**, K43T, **K88Q**, **K88R**, T40I<br>**a514c**, a514t, c462t, c513t, **c517t**<br>-	K43R, K88T, K88R, K88M<br>a514c, a1401g, a906g, c517t, a908c, t1264g, t1322g, g1498t, del -21a<br>D132Y 7 SNP, E92D, L90R, V124A, P38T, G37R, L79W, L79S, L145F, S70R, H48P, Y22C, R47W, A19P, L86F, R96L, L152S, G73A, G34E, del562a, del350c, del179t, del86g
Second-line (group C)	ETO/PTO	*inhA-mabA*<br>*ethA*	**c-15t**, c-15t+I194T, c-15t+S49A<br>-	g-17t, c-15t, T4P, I21T, S94A, V78G<br>A381P, T453I, Q254Stop, A20S, Y32D, ins1332c, R292Stop, Q254P, S390F, ins751c, S55P, S399Stop, W109Stop
Second-line (group D)	Z	*pncA*	t-12c, **a-11g**, t-7c, **A3E**, L4S, **I6T**, **V7G**, D8E, **D8G**, D8N, **Q10P**, Q10R, **D12A**, **D12G**, **D12N**, **C14R**, G17D, L19P, G24D, **Y34D**, **A46V**, **K48T**, **D49G**, D49N, **H51Q**, H51R, **P54L**, P54S, **H57D**[G], H57P, **H57R**, **H57Y**, F58L, S59P, P62L, P62Q, **D63G**, S66P, **S67P**, W68C, **W68G**, **W68R**, **H71D**, H71Q, H71R, **H71Y**, **C72R**, **T76P**, **H82R**, **L85P**, **L85R**, **F94L**, **F94S**, K96E, K96N, K96R, K96T, G97C, **G97D**, **G97S**, **Y103H**, **S104R**, **G108R**, L116P, **L116R**, **L120P**, R123P, V125F, **V125G**, **V128G**, G132A, G132D, G132S, I133T, A134V, **T135N**, **T135P**, H137P, C138Y, **V139A**, **V139G**, **V139L**, **Q141P**, **T142A**, **T142K**, **T142M**, **indel - R148ins (inframe)**, L151S, **V155G**, L159P, T160P, G162D, T168P, A171E, **L172P**, M175I, M175T, **M175V**, V180F, V180G, **Pooled frameshifts and premature Stop codons**	H51R, L120P, Q10P, T135P, P54L, L120R 9 INS, T76P, G97S, Y103Stop, T142A, G132D, Q10H, A134V, G132S, D12G, Q141P, V180F, A146E, V21G, I90T, V9G, H57L, W119G, D49N, A146V, L85P, D12A, I5T, D8N, F58L, T76I, F106S, D136G, W68R, K48T, Q10Stop, T100P, S67Stop, V139A, Q122Stop, L182W, H57Y, I31S, L172P, G108E, Y103H, G97D, L182S, V130E, A171V, L156P, V131G, G78G, Y64Stop, V93G, V130G, G78V, S67P, A102P, H57D, D12E, C14R, Y64Stop, S66P, T153N, P69S, M175R, S67W, K96Q, Y34D, I133T, W68G, D63A, H51P, L19P, V139G, T142P, T142M, D8G, H57R, C72R, T167I, D49E, K96E, V180G, F58C, K96T, T160A, L182F, V155G, T47A, T100I, M175T, H71R, L27P, H71P, V139M, L4S, Q10R, V93L, F81V, D63G, L159R, V125F, H51Y, V128G, t-30c, ins390c, ins354a, t-37a, ins390cc, del302tccggtgtag, del299ggtgta, del465gcaccctg, ins416c, ins 406t, ins232c, ins191t, del544aact, t-30g, del 181gtgccgga, ins299t, del318c, a-31c

References

1. Farhat MR, Sultana R, Iartchouk O, et al. Genetic Determinants of Drug Resistance in *Mycobacterium tuberculosis* and Their Diagnostic Value. *Am J Respir Crit Care Med* 2016; **194**(5): 621-30.
